# Mycothiol Peroxidase Activity as a Part of the Self-Resistance Mechanisms against the Antitumor Antibiotic Cosmomycin D

**DOI:** 10.1128/spectrum.00493-22

**Published:** 2022-05-05

**Authors:** Roger D. Castillo Arteaga, Leandro M. Garrido, Brandán Pedre, Irina Helmle, Harald Gross, Bertolt Gust, Gabriel Padilla

**Affiliations:** a Pharmaceutical Institute, Department of Pharmaceutical Biology, University of Tübingen, Tübingen, Germany; b Institute of Biomedical Sciences, University of São Paulo, São Paulo, Brazil; c Division of Redox Regulation, DKFZ-ZMBH Alliance, German Cancer Research Center (DKFZ), Heidelberg, Germany; d German Center for Infection Research (DZIF), Partner Site Tübingen, Tübingen, Germany; Francis Crick Institute

**Keywords:** self-resistance mechanisms, *Streptomyces olindensis*, cosmomycin D, mycothiol peroxidase, reactive oxygen species

## Abstract

Antibiotic-producing microorganisms usually require one or more self-resistance determinants to survive antibiotic production. The effectors of these mechanisms are proteins that inactivate the antibiotic, facilitate its transport, or modify the target to render it insensitive to the molecule. Streptomyces bacteria biosynthesize various bioactive natural products and possess resistance systems for most metabolites, which are coregulated with antibiotic biosynthesis genes. Streptomyces olindensis strain DAUFPE 5622 produces the antitumor antibiotic cosmomycin D (COSD), a member of the anthracycline family. In this study, we propose three self-resistance mechanisms, anchored or based in the COSD biosynthetic gene cluster. These include *cosIJ* (an ABC transporter), *cosU* (a UvrA class IIa protein), and a new self-resistance mechanism encoded by *cosP*, which shows response against peroxides by the enzyme mycothiol peroxidase (MPx). Activity-based investigations of MPx and its mutant enzyme confirmed peroxidation during the production of COSD. Overexpression of the ABC transporter, the UvrA class IIa protein, and the MPx led to an effective response against toxic anthracyclines, such as cosmomycins. Our findings help to understand how thiol peroxidases play an antioxidant role in the anthracycline producer S. olindensis DAUFPE 5622, a mechanism which has been reported for neoplastic cells that are resistant to doxorubicin (DOX).

**IMPORTANCE** Anthracycline compounds are DNA intercalating agents widely used in cancer chemotherapeutic protocols. This work focused on the self-resistance mechanisms developed by the cosmomycin-producing bacterium Streptomyces olindensis. Our findings showed that cysteine peroxidases, such as mycothiol peroxidase, encoded by the gene *cosP*, protected S. olindensis against peroxidation during cosmomycin production. This observation can contribute to much better understanding of resistance both in the producers, eventually enhancing production, and in some tumoral cell lines.

## INTRODUCTION

Anthracyclines are part of the polyketide type II family and are an important group of natural compounds produced by actinobacteria. The basic structure contains an aglycone skeleton and 6-deoxyhexose moieties, biosynthetically derived from glucose-1-phosphate. The two anthracyclines reported first were isolated from the pigment producer Streptomyces peucetius and were named doxorubicin (DOX) and daunorubicin (DNR) (1). Anthracyclines are clinically important compounds and are widely used to date for the treatment of different cancers, such as breast cancer, lymphomas, acute leukemia, neuroblastomas, and bone and soft tissue sarcomas (2–4). Streptomyces olindensis strain DAUFPE 5622 produces a purple-pigmented anthracycline with both antimicrobial activity (e.g., a MIC of 0.01 μg/mL against Staphylococcus aureus strain ATCC 29213) and antitumoral activity (e.g., a 50% inhibitory concentration [IC_50_] of 0.110 μg/mL against the HeLa human cervical carcinoma cell line) (5). Structural studies determined that the molecule, called cosmomycin D (COSD), is an aromatic complex with two trisaccharide chains attached at positions C-7 and C-10 on the aglycone (5, 6). Cosmomycins are an interesting group of compounds because they show one of the most complex glycosylation patterns found in anthracyclines, with position C-10 less frequently glycosylated than position C-7 (7).

Antitumor-antibiotic-producing microorganisms must be protected from the lethal effects of their own products. The ability inherent in the producer organisms is called self-resistance. An understanding of the underlying mechanisms of drug resistance of pathogenic bacteria and self-resistance mechanisms of antitumor-antibiotic-producing microorganisms is important to develop therapeutic drugs for the treatment of infectious diseases (8, 9). Antibiotic-producing microorganisms have a system that leads to transcription of the antibiotic-biosynthesizing genes at an appropriate time. To act effectively for self-resistance to its own antibiotic, antibiotic-modifying enzymes, target-protecting enzymes, antibiotic excretion systems, or DNA repair mechanisms must be present in the producer organisms when the biosynthetic pathway begins to produce the antibiotic (9–11).

The resistance mechanisms found in some anthracycline producers, such as S. peucetius, occur by the action of DrrA and DrrB, members of the ABC family of membrane transporters, which influence the efflux of DOX and DNR by the formation of an ABC transporter complex comprised of an ATPase and a transmembrane protein (10, 12–14). The second mechanism involves an inhibition or destabilization of the binding of DOX and DNR to genomic DNA by the action of the gene product of *drrC*, which codes for a protein with similarity to bacterial UvrA (10, 13–15).

In comparison with other anthracyclines, COSD exhibits a high toxicity (16), which leads to the hypothesis that this strain must possess additional or optimized self-resistance strategies. In the biosynthetic gene cluster (BGC), we identified a gene encoding a mycothiol peroxidase (MPx) inside the cosmomycin cluster, adjacent to other resistance genes that were very similar to those reported in other actinobacteria that use the low-molecular-weight thiol mycothiol (MSH) (17). MPx is a thiol peroxidase that belongs to the cysteine glutathione peroxidase (CysGPx)-like enzyme family, having the S-mycothiolation of the MPx as part of its H_2_O_2_ catalytic mechanism involved in controlling the reactive oxygen species (ROS) levels (17). There are two MPx paralog genes in the S. olindensis genome, while other species, such as Streptomyces coelicolor and Streptomyces avermitilis, have only one MPx.

We observed for the first time that MPx can reduce a cysteine–MSH mixed disulfide, using a dithiol disulfide exchange mechanism, during the biosynthesis of COSD. Purification and identification of the MPx protein provided evidence that ROS-detoxifying proteins are employed by anthracycline producers. We describe in this study three self-resistance mechanisms against COSD in S. olindensis. The genes *cosI* and *cosJ* encode the first mechanism, an ABC transporter efflux system, while the second mechanism, encoded by *cosU*, is similar to *drrC* of S. peucetius and UvrA class IIa homologue proteins. Finally, a novel third mechanism, a mycothiol peroxidase (MPx) encoded by *cosP*, is involved in the detoxification of H_2_O_2_.

## RESULTS

### Self-resistance genes allocated within the cosmomycin D cluster.

Analysis of the COSD biosynthetic gene cluster, located at contig 2 of the genome assembly of S. olindensis (JJOH00000002.1), enabled the assignment of each gene based on their homology to genes coding for proteins of known functions. The genes *cosI* (DF19_23560), *cosJ* (DF19_23565), *cosP* (DF19_23570), and *cosU* (DF19_23575) were identified as four candidate genes that might play a role concerning self-resistance. The products of *cosI* and *cosJ* are part of a superfamily of transport proteins (ABC transporters) that is one of the largest groups of proteins in nature (18). In detail, *cosI* codes for a nucleotide-binding domain (NBD). The amino acid sequence encoded by *cosI* shows the same features found in the prototypal anthracycline resistance protein DrrA from S. peucetius, such as the Walker A motif, Q loop, signature motif, Walker B motif, switch region, GATE (glycine loop and transducer element) domain, and DEF motif (19). This component is associated with transmembrane proteins that are responsible for the formation of pores through which substances are transported, known as transmembrane domains (TMDs) (20). The analysis of the product of *cosJ* shows a typical conformation of the TMD, composed of alpha helices organized as homodimers (21). Both proteins form part of the ABC transporter complex, which has 95.14% identity to the ABC transporter of Streptomyces purpurascens (accession numbers WP_189725841.1 and WP_189725842.1, respectively) (Fig. S2 in the supplemental material). For CosJ, six transmembrane helices, forming a transmembrane domain, can be predicted using Phyre and TMHMM (Fig. S1).

In close proximity to *cosI* and *cosJ*, *cosP* could be identified as another plausible resistance determinant. It encodes an enzyme (accession number KDN80073.1) that shows 88.34% identity on the amino acid level with the glutathione peroxidase (GPx) from S. purpurascens (accession number GHA30648.1). Although proteins of this kind are commonly annotated as glutathione peroxidases, those in actinobacteria in fact represent mycothiol peroxidases, as enzymatically demonstrated in the protein with accession number ASW14906.1 from Corynebacterium glutamicum (17).

Besides the gene encoding KDN80073.1, there is a second gene annotated with this function in the genome, with locus tag DF19_33265, which is found in contig 4 (JJOH00000004.1) and encodes the protein with accession number KDN79115.1. When we compared both proteins with the MPx of C. glutamicum (accession number ASW14906.1), KDN79115.1 showed a higher identity level (53.21%) than CosP (KDN80073.1) (43.4%), which resulted in higher values for the BLAST score and E value, which were, respectively, 175 and 2e−57 for KDN79115.1 and 139 and 3e−43 for KDN80073.1. When KDN80073.1 and KDN79115.1 were compared, they shared 55.1% identity.

Searching selected Streptomyces reference genomes for annotated GPx or MPx genes, we found in general only one ortholog per genome, such as those that encode the proteins with accession numbers CAB88451.1 in S. coelicolor strain A3(2), WP_010985209.1 in S. avermitilis, WP_100106014.1 in S. peucetius subsp. *caesius* strain ATCC 27952, and EFL32854.1 in Streptomyces viridochromogenes strain DSM 40736 (Fig. S3). To understand how both annotated peroxidases of S. olindensis correlate with these proteins and with the remaining members of the GPx family in Pfam (accession number PF00255), similarity sequence networks (SSNs) were constructed.

Since KDN80073.1 was not in the UniProt database, first a small SSN with 100 proteins, denominated “seed,” was generated with the Enzyme Similarity Tool (22) and the FASTA sequence of KDN80073.1 (Fig. S5), with the aim of identifying close relatives of KDN80073.1 within the GPx family in Pfam. Since an identity level of 60% or higher was assumed to be sufficient to identify isofunctional protein clusters (22), we chose a level of 70% identity as the threshold in the seed SSN, where cluster 2 contained KDN80073.1 and a further 12 members, listed in Table S2.

A first SSN of Pfam family PF00255 was obtained using a threshold of about 55% similarity (Fig. S6); a large main cluster of 39,799 peroxidases included almost all actinobacterial proteins, together with those from other phyla, including known prokaryotic (e.g., BtuE of Escherichia coli [accession number P06610]) and eukaryotic (e.g., GPx1 of Saccharomyces cerevisiae [accession number P36014]) GPx proteins. When the threshold was increased to a similarity level of about 61% in a second PF00255 SSN, the actinobacterial proteins grouped in different clusters. Cluster 2 comprised 3,261 proteins and included the proteins with accession numbers CAB88451.1, WP_010985209.1, WP_100106014.1, and EFL32854.1; however, cluster 99 had almost the same composition as seed SSN cluster 2, excluding only KDN80073.1 (Fig. S7 and Table S2), which is not in the PF00255 database.

Subsequently, a genome neighborhood network (GNN) was generated from that SSN (Fig. S8). The analysis of cluster 99 revealed that all members were in non-previously described cosmomycin clusters of different Streptomyces species. These findings could suggest that KDN80073.1 has an increased specificity for some peroxides generated by cosmomycin action.

The third putative resistance mechanism is encoded by *cosU*, whose product shows 96.71% similarity to the excinuclease ABC subunit UvrA of Streptomyces janthinus (accession number WP_193483046) (Fig. S4), which is a member of a group of proteins associated with DNA repair (10, 23). The prototype of this group is DrrC from S. peucetius, which could bind to daunorubicin intercalated to DNA and displace anthracycline, thereby preventing nucleic acid damage and allowing the drug to be expelled by the DrrA/-B system (23).

### Self-resistance mechanisms are essential for *S. olindensis* when challenged with DOX and COSD.

**FIG 1:**
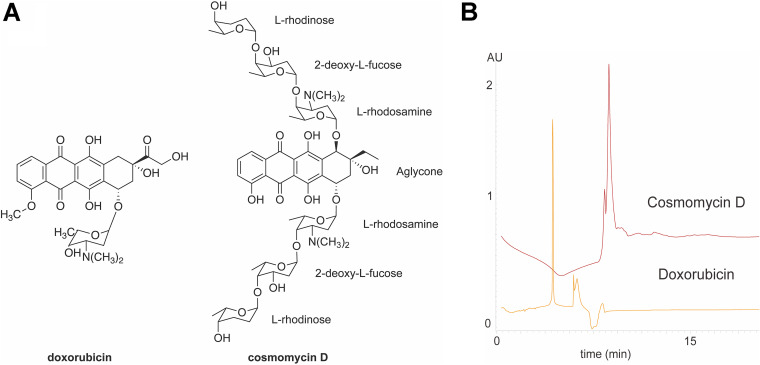
(A) Chemical structures of doxorubicin (DOX) and cosmomycin D (COSD). (B) HPLC profiles of COSD and DOX.

To understand the toxicity of COSD compared with that of pure DOX, COSD was isolated from S. olindensis using analytical fractionation. The fraction of interest showed a red-purple color. High-resolution mass spectrometry (HR-MS) measurements revealed a candidate compound with protonated molecular masses of *m/z* 1,189.5901 ([M + H]^+^, calculated 1,189.590149, Δ = −0.04 ppm) and *m/z* 595.2991 ([M + 2H]^2+^, calculated 595.298713, Δ = +0.65 ppm), which were consistent with the molecular formula of COSD (C_60_H_88_N_2_O_22_) (Fig. 1). A complementary tandem mass spectrometry (MS/MS) analysis of COSD, executed on its [M + 2H]^2+^ ion, produced fragment ions that were in full agreement with the expected structure (Fig. S9 and Table S1).

**FIG 2:**
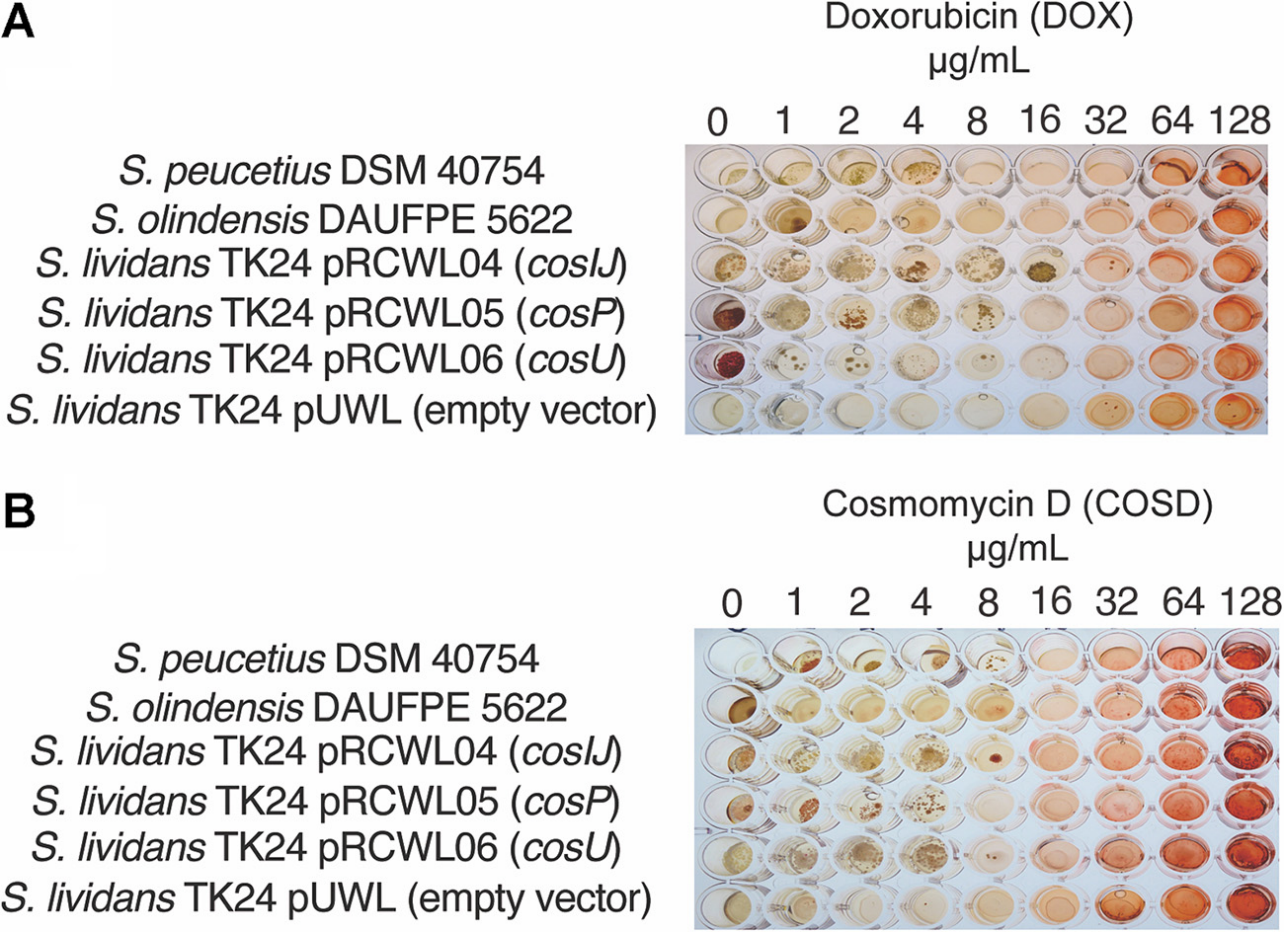
MICs of COSD and DOX for selected Streptomyces species by 96-well-plate assay. S. peucetius DSM 40754, S. olindensis DAUFPE 5622, S. lividans TK24/pRCWL04 (*cosI cosJ*), S. lividans TK24/pRCWL05 (*cosP*), S. lividans TK24/pRCWL06 (*cosU*), and S. lividans TK24/pUWL (empty vector) were challenged with DOX (A) or COSD (B). Final concentrations of DOX and COSD were 0, 1, 2, 4, 8, 16, 32, 64, and 128 μg/mL for both antibiotics (apramycin was added at 50 μg/mL when needed).

The hypothetical self-resistance genes *cosI*, *cosJ*, *cosP*, and *cosU* were cloned into the expression plasmid pUWL_Apra_oriT, generating the recombinant constructs pRCWL04 (*cosI cosJ*), pRCWL05 (*cosP*), and pRCWL06 (*cosU*). Exconjugants of S. lividans strain TK24 carrying the corresponding constructs, as well as the empty vector, were used to determine the MICs (Table 1) in a 96-well-plate assay format against COSD and doxorubicin (DOX) (Fig. 2). As controls, S. olindensis and S. peucetius were used.

**TABLE 1:** MICs of doxorubicin and cosmomycin D against Streptomyces strains

Strain	MIC (μg/mL) of:	
	Doxorubicin	Cosmomycin D
S. peucetius DSM 40754	4	4
S. olindensis DAUFPE 5622	4	8
S. lividans TK24/pRCWL04 (*cosIJ*)	16	8
S. lividans TK24/pRCWL05 (c*osP*)	8	4
S. lividans TK24/pRCWL06 (*cosU*)	16	8
S. lividans TK24/pUWL (empty vector)	2	2

The endogenous resistance to COSD of S. lividans TK24 was 2 μg/mL; nevertheless, when it harbored the *cosI* and *cosJ* genes or the *cosU* gene, the resistance to COSD increased to the same level as that of S. olindensis (8 μg/mL). The recombinant containing the mycothiol peroxidase (*cosP*) did not reach this concentration, but it hit the concentration achieved by S. peucetius strain DSM 40754 (4 μg/mL). In a similar pattern, the *cosI* and *cosJ* genes or the *cosU* gene conferred higher resistance to DOX (16 μg/mL) than did *cosP* (8 μg/mL).

S. olindensis had a resistance to DOX similar to that of S. peucetius; however, for COSD, S. olindensis was more resistant (8 μg/mL versus 4 μg/mL). On the other hand, S. lividans TK24 containing the empty pUWL vector did not show survival when exposed to COSD and DOX concentrations higher than 1 μg/mL.

### Self-resistance genes are mostly expressed during the production of COSD.

**FIG 3:**
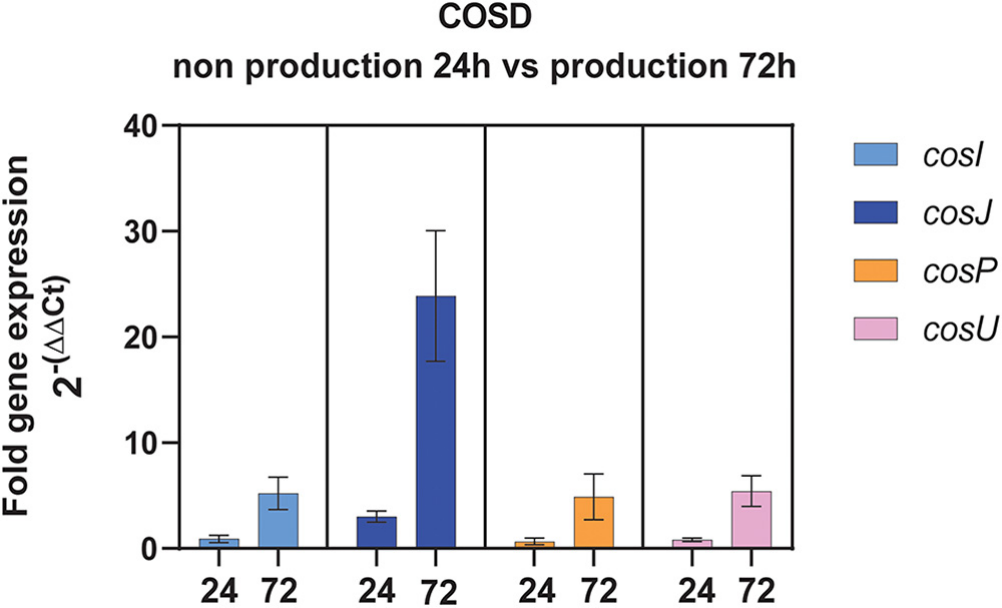
qPCR of *cosI*, *cosJ*, *cosP*, and *cosU*, with *hrdB* as the reference, during production (P) (72 h of cultivation) and nonproduction (N) (24 h of cultivation) of COSD by S. olindensis. Values are means of triplicate determinations ± standard deviations (SD).

We hypothesized that the S. olindensis wild-type (WT) strain overexpresses its self-resistance genes during production of the antibiotic. Thus, the expression levels of these genes were determined by quantitative PCR (qPCR) both during COSD production at 72 h and when COSD production was not detectable, at 24 h of cultivation (6). The endogenous gene *hrdB* (24) served as the reference (Fig. S10). For all genes, it was possible to confirm overexpression during the time when cosmomycin was produced compared with their expression levels during the cultivation stage when COSD was not yet detected (Fig. 3).

### MPx response against ROS stress.

**FIG 4:**
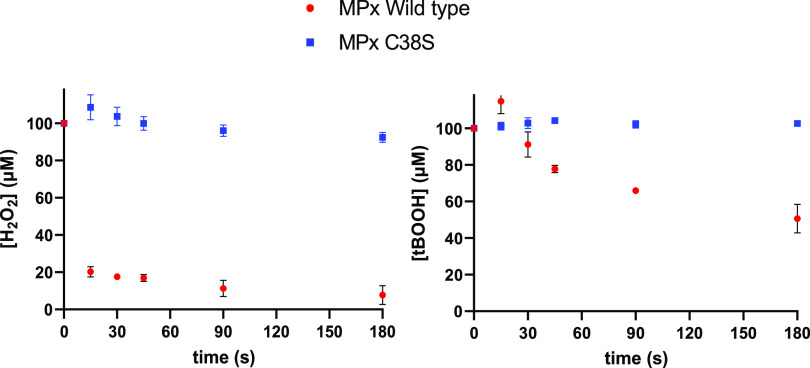
H_2_O_2_ and tBOOH quantification (FOX assay) of MPx WT and MPx C38S during 180 s of activity. Values are means of triplicate determinations ± SD.

The mycothiol peroxidase (MPx) from S. olindensis consists of 163 amino acids, three of which are Cys residues (C38, C66, and C84). Additionally, we purified a C38S mutant enzyme of MPx (bearing a mutation of Cys to Ser at position 38), obtained by site-directed mutagenesis (Fig. S11). We evaluated the peroxidase activity of both enzymes by ferrous oxidation of xylenol orange (FOX) assay to quantify H_2_O_2_ and *t*-butyl hydroperoxide (tBOOH) reduction within a time frame of 180 s. The WT MPx consumed the majority of H_2_O_2_ within 15 s, whereas the C38S mutant enzyme remained catalytically inactive, demonstrated by a constant amount of H_2_O_2_ remaining in the assay over the time used. The FOX assay performed with tBOOH showed a significant decrease in the concentration of tBOOH, although the consumption rate was slower than in the case of H_2_O_2_, as only half of the tBOOH was reduced after 180s (Fig. 4).

**FIG 5:**
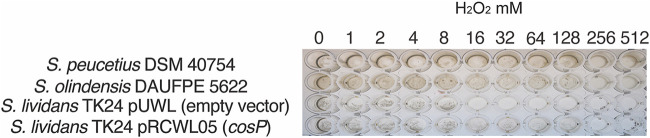
MICs for selected Streptomyces species against H_2_O_2_ in a 96-well-plate assay. S. peucetius DSM 40754, S. olindensis DAUFPE 5622, S. lividans TK24/pRCWL05 (*cosP*), and S. lividans TK24/pUWL (empty vector) were challenged with H_2_O_2_ at the indicated concentrations.

We also evaluated the MICs of H_2_O_2_ against S. olindensis, S. peucetius, and S. lividans (1 to 512 mM). For the concentrations tested, both of the anthracycline producers S. olindensis and S. peucetius survived 128 mM H_2_O_2_, thereby confirming a good response against H_2_O_2_. S. lividans was very sensitive to H_2_O_2_ (MIC of 8 mM) (Table 2), but its resistance to H_2_O_2_ improved notably when it contained a vector expressing *cosP*, showing a MIC of 32 mM (Fig. 5 and Table 2). This result indicates that MPx relieves this stress on S. olindensis, allowing it to survive persistent oxidative conditions.

**TABLE 2:** MICs of H_2_O_2_ against Streptomyces strains

Strain	MIC (mM) of H_2_O_2_
S. peucetius DSM 40754	128
S. olindensis DAUFPE 5622	128
S. lividans TK24/pUWL (empty vector)	8
S. lividans TK24/pRCWL05 (*cosP*)	32

### *S. olindensis* peroxidase detoxification is important for survival during cosmomycin D production.

**FIG 6:**
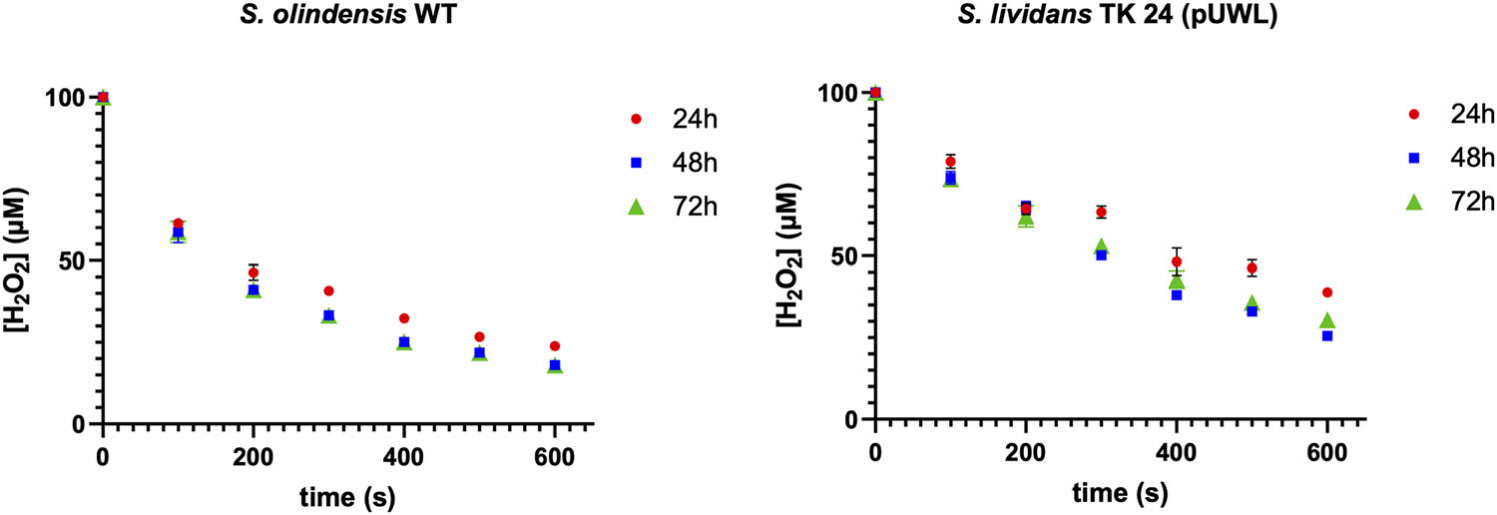
H_2_O_2_ quantification (FOX assay) of S. olindensis WT versus S. lividans TK24 during 600 s of activity. Values are means of triplicate determinations ± SD.

To understand the role of CosP in S. olindensis H_2_O_2_ consumption, we evaluated the capacity of COSD producer S. olindensis and nonproducer S. lividans to reduce H_2_O_2_ over time, under conditions that favor COSD production. For this, we measured the rates of H_2_O_2_ consumption of total protein extracts at 24, 48, and 72 h of growth (Fig. 6). While both S. olindensis and S. lividans improved their H_2_O_2_ consumption rates at later growth stages, it was obvious that the *cosP* producer S. olindensis reduced H_2_O_2_ faster (Fig. 6).

## DISCUSSION

In this work, we highlighted that S. olindensis has an extra MPx copy encoded by the cosmomycin biosynthetic gene cluster (BGC), acting in complementarity with the previously studied ABC transporter and UvrA class IIa anthracycline resistance proteins as a possible self-resistance factor during the production of COSD. Since other anthracycline BGCs containing the same genes are described in the literature—for example, a cytorhodin BGC (accession number MF773975.1) and other BGCs listed in Fig. S8—our findings can most likely be extended to some anthracycline-producing bacteria.

The protein encoded by *cosI* belongs to the superfamily of ATP-dependent ABC transporters. The organization of this protein includes two motifs, Walker A and Walker B, providing an ATP-binding site, and contains the unique signature motif in ABC transporters that is located upstream from the Walker B motif. For S. olindensis, it was classified as type I: the transporter system consists of two proteins encoded by independent genes, with CosI (NBD) containing the nucleotide-binding domains (Walker A and Walker B) and CosJ (TMD), the hydrophobic membrane protein, containing six transmembrane (TM) helices (Fig. S1).

ABC transporters belonging to this class have been so far reported solely in organisms that are producers of several antitumor agents (25). Daunorubicin and doxorubicin are anthracycline drugs produced by S. peucetius. Two genes (*drrA* and *drrB*) whose products form a type I transporter system have been cloned and found to confer resistance to daunorubicin/doxorubicin and mithramycin (*mtrA* and *mtrB* genes) (13, 14, 26–29).

In Gram-positive bacteria, such as actinobacteria, it has been reported that MPx is a novel CysGPx peroxidase family that degrades hydrogen peroxide and alkyl hydroperoxides in the presence of either the thioredoxin/thioredoxin reductase (Trx/TrxR) or mycoredoxin 1/mycothiol/mycothione reductase (Mrx1MSH/Mtr) reducing systems. MPx protects against the damaging effects of ROS induced by multiple stressors, using thioredoxin or related redoxins as reductants (17, 30, 31). The S. olindensis genome has two putative genes that code for peroxidase-type proteins (accession numbers KDN80073.1 and KDN79115.1) and are involved in the general detoxification of H_2_O_2_. The protection is not only related to anthracycline production, in agreement with the results shown in Fig. 5 for the challenge with H_2_O_2_, in which S. olindensis and S. peucetius show a good detoxification response that is not related to any type of response against anthracycline production. Despite S. olindensis possessing two proteins that could cooperate in ROS depuration, the activity against H_2_O_2_ almost disappears in the KDN80073.1 mutant, indicating that this protein is an important scavenger of this peroxide in the cell, while S. peucetius has just one MPx annotated in its genome; however, it is more closely related to KDN79115.1 (67.82% identity) than to KDN80073.1 (56.33% identity).

It is known that anthracyclines produce free radicals that may, in association with ferric compounds, cause oxidative stress (32). Oxidative stress depends on reactive oxygen species (ROS), which are generated by the action of xenobiotics and because of metabolic processes (33). The oxidizing power of O_2_^.−^ and H_2_O_2_ is harmful to cells. These species can inactivate important metabolic enzymes or alter their catalytic activity. H_2_O_2_ can cross the cell membrane and initiate lipid oxidation by deprotonation of fatty acids (34).

Biochemical and physiological data lead to the hypothesis that anthracyclines like DOX or, in our case, COSD can cause the formation of free radicals that stimulate lipid peroxidation and alter cellular integrity (35, 36). This hypothesis supports the premise that the oxidative metabolism of the anticancer quinones represents a significant contribution to the cytotoxic effects of these compounds (37). These species can inactivate important metabolic enzymes, altering their catalytic activity. In the case of H_2_O_2_, it can cross the cell membrane and initiate lipid oxidation by deprotonation of fatty acids (34), similar to findings reported for DOX during the redox cycle of anthracyclines (38).

Interestingly, the study of Westman and collaborators (39) correlated the biological activity of 7-deoxydoxorubicinolone in prokaryotes and eukaryotes. The authors could show that the levels of expression of catalase, superoxide dismutase, and glutathione peroxidase in bacterial cells are higher than the levels in human cells from cardiac tissue. They concluded that bacterial cells are much more competent at dealing with the outcomes of anthracycline semiquinone oxidation-reduction cycling and the resulting reactive oxygen species (ROS) that evolve and that Streptomyces bacteria are generally well equipped to deal with reactive oxygen stress (39). The aglycone molecules may accumulate in membranes due to their hydrophobicity; the rapid growth and division of bacterial cells relative to that of eukaryotic cardiac tissue may also explain the differential effects of anthracycline aglycones on prokaryotes and eukaryotes (39).

Based on our present findings, we hypothesize that anthracyclines, such as COSD, are determinant in the production of ROS and that the recruitment of additional antioxidant enzymes coded in BGCs, such as KDN80073.1, can increase the self-resistance of antitumor antibiotics with potential cell toxicity. Another line of evidence is that KDN80073.1 groups with an independent protein cluster in the Pfam PF00255 family SSN, wherein all members belong to cosmomycin-like clusters (Fig. S7), which could also indicate that these proteins boost the protection against anthracycline-generated intracellular ROS.

COSD reportedly binds DNA more tightly than DOX (40), but it causes less DNA damage than DOX; even so, the levels of apoptosis induced by both drugs in nucleotide excision repair-deficient fibroblasts are similar (16). CosU is responsible for minimizing the interaction between COSD and DNA, thereby avoiding damage.

Normally, DNA damage recognition is performed by the protein UvrA, which belongs to the ABC ATPase superfamily. Bacterial UvrA is a dimeric protein, unique among DNA repair enzymes. This feature enables UvrA to detect various DNA lesions by using an indirect readout mechanism. Other proteins, namely, UvrB, UvrC, and UvrD, are components of nucleotide excision repair (NER) and are essential for the survival of almost every living bacterium (41, 42).

The protein encoded by the *cosU* gene has a high similarity to UvrA proteins; however, it contains deletions of the UvrB-binding domain and the first zinc finger motif. For this reason, the protein function is not involved in NER and it is classified as a UvrA-like class IIa protein (43). Proteins of the latter class have been shown to behave *in vitro* like ATP-dependent DNA-binding proteins, such as that of S. peucetius from the daunorubicin cluster (10, 15), the nogalamycin produced by Streptomyces nogalater (44), the product of *cmrX* that confers resistance to chromomycin in Streptomyces griseus subsp. *griseus* (45), and the product of *mtrX*, which is a UvrA-like protein involved in mithramycin resistance in Streptomyces argillaceus (46).

Interestingly, the most common characteristic of these antitumor antibiotics is that they intercalate to DNA and could represent a DNA-binding protein that plays a role in self-resistance, inhibiting or destabilizing the interaction of these drugs with genomic DNA, thus preventing intercalating antibiotics from interfering with cell transcription and/or replication. This suggests in turn that the function of UvrA class IIa proteins is associated with the removal of noncovalent DNA-binding agents.

**FIG 7:**
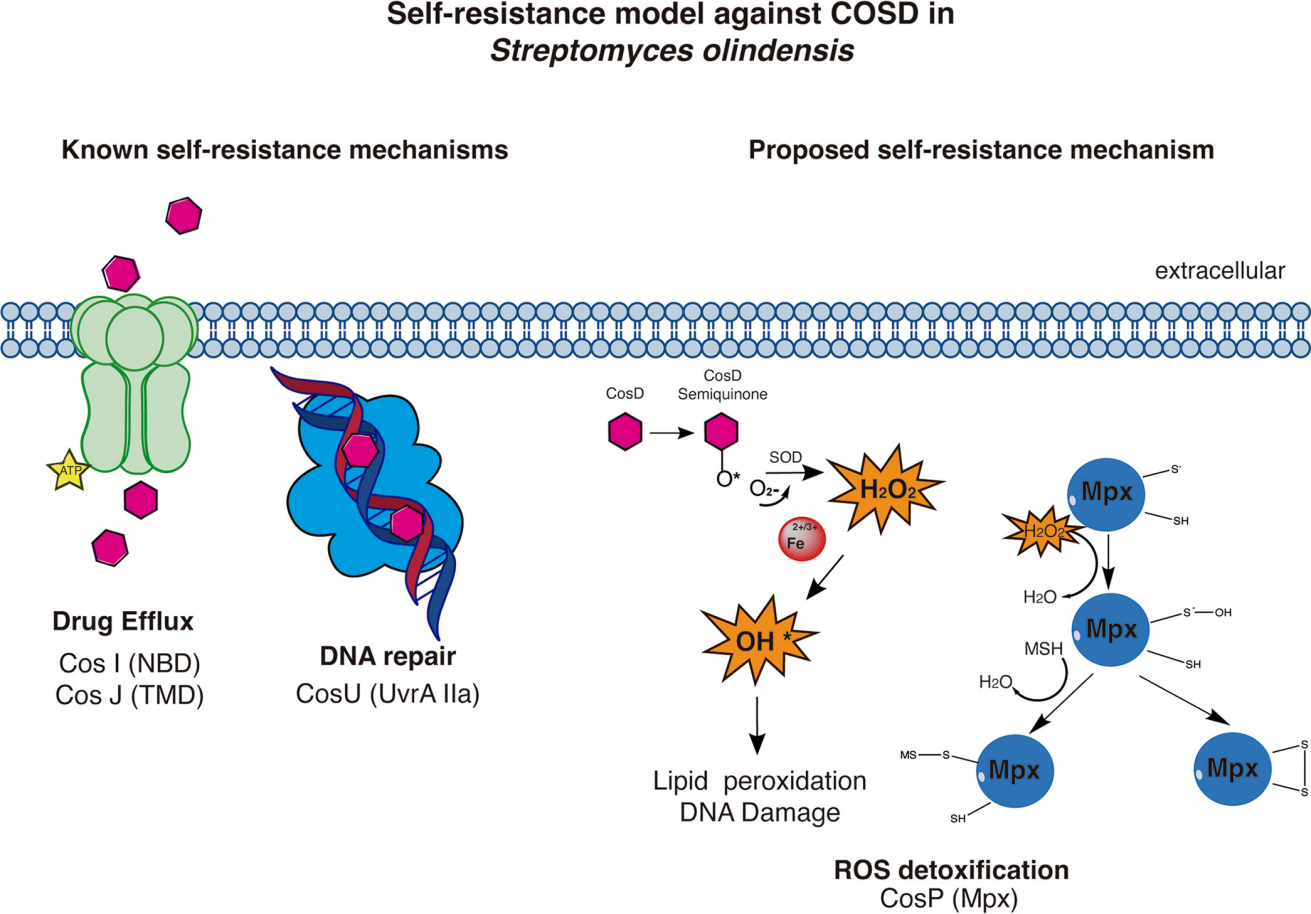
Proposed model for self-resistance to cosmomycin D in S. olindensis. Mechanisms of self-resistance for COSD: drug efflux function is by CosI and CosJ; CosU protein is involved in DNA repair, scanning the COSD-DNA-binding complex; and CosP (MPx) enzyme is proposed as a new self-resistance mechanism for H_2_O_2_ detoxification.

Finally, we propose a model (Fig. 7) for the self-resistance in S. olindensis strain DAUFPE 5622 during cosmomycin D biosynthesis. This compound is recognized by the ABC transporter CosI/CosJ, and then mycothiol peroxidase (CosP) participates in the protection against H_2_O_2_ and lipid peroxidation caused by anthracyclines. The UvrA-like protein is important for reducing the interaction between DNA and the antibiotic. This mode of resistance represents an advantage for the producer strain for survival during antibiotic biosynthesis; the organism avoids the interaction of the harmful compound COSD with its intracellular target. Streptomyces evolution developed different strategies of self-resistance, and these depend on the form of action of the active compound. It is important to understand those mechanisms in producer microorganisms to evaluate plausible horizontal transfer in pathogenic strains and to develop therapeutic alternatives.

## MATERIALS AND METHODS

### Bacterial strains and culture conditions.

Strains used for cloning or conjugation were E. coli XL11 blue (Agilent technologies) and the methylation-deficient E. coli ET12567/pUZ8002 (47), respectively. E. coli strains were grown and maintained at 37°C in Luria-Bertani (LB) medium or LB agar (LB with 1.5% agar) supplemented with 30 mg/mL chloramphenicol, 50 mg/mL kanamycin, and 50 mg/mL apramycin, when required.

Streptomyces strains were obtained from stocks of the actinobacteria collection of the pharmaceutical biology department of the University of Tübingen. S. olindensis DAUFPE 5622 was obtained through G. Padilla from the Institute of Biomedical Sciences of the University of Sao Paulo. S. peucetius DSM 40754 was obtained from the German Collection of Microorganisms and Cell Cultures GmbH (DSMZ).

Streptomyces strains were grown and maintained at 30°C in trypticase soy (TS) broth and/or TS agar (Sigma-Aldrich), mannitol-soy agar (2% mannitol, 2% soybean meal, 10 mM MgCl_2_, 1.5% agar), R2YE (48), and R5M to produce cosmomycin D (5).

### Cosmomycin D production, purification, and structure integrity.

Doxorubicin was obtained from Sigma-Aldrich. Cosmomycin D utilized in this study was isolated and purified from 5-L R5M medium fermentations of S. olindensis, as previously reported (6).

For analysis of cosmomycin production, the crude extract was dissolved in methanol (liquid chromatography-mass spectrometry [LC-MS] grade; Merck) and applied to a solid-phase extraction cartridge (Strata-XL, 100 μm polymeric reversed phase, 2 g; Phenomenex). The adsorbed compounds were eluted in fractions using a stepwise gradient of 10% to 100% methanol and concentrated under reduced pressure. After resuspension of the residue, 10-μL samples were analyzed by high-performance (HP) LC-MS, using a reverse-phase column (Luna Omega Polar C_18_, 3 μm, 150 mm by 3.0 mm; Phenomenex) and a mobile phase of acetonitrile (solvent A) and 0.06% formic acid in water (solvent B). A gradient of 10% B to 100% B in 22 min, ending with 100% B for an additional 8 min, with a flow rate of 0.2 mL/min was used. The fraction containing COSD (*m/z* 1,189.54) was further purified using a Waters HPLC system (Waters 1525 binary pump with a 7725i Rheodyne injection port, a Kromega solvent degasser and a Waters 996 photodiode array detector), using a linear gradient of 40% B to 60% B in 15 min, followed by a linear gradient of 60% B to 95% B in 20 min and an additional 10 min at 95% B (solvent A, 0.06% formic acid in water; solvent B, 0.1% formic acid in acetonitrile) at a flow rate of 2 mL/min (Luna Omega Polar C_18_ column, 5 μm, 250 mm by 4.6 mm; Phenomenex) to obtain ~5 mg pure COSD.

For structure determination, high-resolution LC-electrospray ionization (ESI)/MS and MS/MS measurements were performed on a Bruker Daltonics maXis 4G (Bruker Daltonics, Bremen, Germany) connected to a Thermo Scientific UltiMate 3000 system (Thermo Fisher Scientific), using a reversed-phase Luna Omega Polar C_18_ column (3 μm, 150 by 3 mm) at a flow rate of 0.3 mL/min. A linear gradient of 10% to 100% solvent B in 40 min, ending with 100% B for an additional 15 min, was used (solvent A, 0.1% formic acid in water; solvent B, acetonitrile), with an injection volume of 5 μL and UV monitoring occurred at 210, 254, 280, and 360 nm. The range of MS acquisition was *m/z* 50 to 1,800. The acquisition parameters for the positive ion polarity were a capillary voltage of 4.5, nebulizer gas pressure (nitrogen) set to 2.0 bar, and dry gas flow of 9.0 L/min at an ion source temperature of 200°C. The measurements were internally calibrated using sodium formate as a reference. The data were compared to data of cosmomycin D and to published data of various related compounds (Fig. S9, Table S1) (6, 16).

### DNA isolation and manipulation.

Plasmid preparation from E. coli was carried out with a commercial kit (QIAprep spin miniprep kit; Qiagen). Total Streptomyces DNA isolation, restriction endonuclease digestions, and ligations were performed as described previously (48, 49). DNA sequencing was performed at Eurofins Genomics Germany GmbH.

### Cloning and construction of plasmids for heterologous expression of cosmomycin resistance genes in S. lividans TK24.

*cosI*, *cosJ*, *cosP*, and *cosU* genes were amplified from S. olindensis DAUFPE 5622 genomic DNA using Phusion high-fidelity DNA polymerase from New England Biolabs, Inc., according to the manufacturer’s instructions, using the primers listed in Table S3. The resulting PCR products were cloned into the pUWL_Apra-oriT vector containing an apramycin resistance gene cassette under the control of the constitutively active ermE* promoter to ensure a high level of expression. These constructs (Table S4) were transferred into E. coli XL1 blue and further into E. coli ET12567/PUZ8002 to obtain nonmethylated DNA. The constructs pRCWL04 (*cosI* and *cosJ*), pRCWL05 (*cosP*), and pRCWL06 (*cosU*) were finally transferred into S. lividans TK24 by conjugation (50).

### Expression and purification of recombinant CosP and CosP(C38S) in E. coli.

For cloning and expression of the mycothiol peroxidase CosP, the IPTG (isopropyl-β-d-thiogalactopyranoside)-inducible vector pHIS8 (51) was used to generate an N-terminally His_8_-tagged CosP. *cosP* was amplified by PCR from genomic DNA (gDNA) of S. olindensis using primers containing recognition sequences for EcoRI and HindIII, respectively. The PCR product was cloned into the expression vector through ligation, taking advantage of the EcoRI*/*HindIII restriction sites, generating plasmid pRCWL04. The integrity of the resulting plasmids was verified via sequencing.

Plasmid pRCWL04 was further modified in the CALA coding region of the enzyme (MPx), exchanging cysteine (C) for serine (S) at position 38, using the QuikChange II site-directed mutagenesis kit (Agilent Technologies), resulting in plasmid pRCWL04M.

Heterologous expression and purification of His_8_-tagged proteins were carried out using E. coli Rosetta 2(DE3)pLys (Novagen, Darmstadt, Germany) as the recombinant host strain. Strains containing the corresponding plasmid were cultivated in 1 L LB broth supplemented with 25 μg/mL chloramphenicol and 50 μg/mL kanamycin at 37°C and 220 rpm. At an optical density at 600 nm (OD_600_) of 0.6, the temperature was adjusted to 18°C and IPTG was added to a final concentration of 0.5 mM.

The overnight culture was harvested by centrifugation at 8,000 rpm and 4°C for 10 min. The supernatant was discarded, and the pellet resuspended in lysis buffer (3 mL/g pellet) (50 mM Tris-HCl, pH 8, 500 mM NaCl, 10% glycerin [vol/vol], 1% Tween 20 [vol/vol], 20 mM imidazole, 10 mM β-mercaptoethanol, 0.5 mg/mL lysozyme, 0.5 mM phenylmethylsulfonyl fluoride [PMSF], and 4 mg/mL lysozyme). The cells were sonicated (Sonifier generator; Branson) on ice for 10 min (amplitude of 40%, 5 s on/5 s off). After centrifugation for 45 min at 18,000 rpm (4°C), the clear lysate was filtered (0.45 μm) for affinity chromatography.

Supernatant of each culture was applied to affinity chromatography using an Äkta start platform (GE Healthcare) equipped with a 5-mL His-Trap HP column (GE Healthcare). The His-tagged protein was washed and then eluted from the column using a linear gradient of 0 to 100% elution buffer over 60 min and collected by a Frac30 system (GE Healthcare). Fractions were tested for the presence of the respective proteins by SDS-PAGE and were concentrated and buffer exchanged into 25 mM KH_2_PO_4_,100 mM NaCl, pH 8.3, using an Amicon Ultra centrifugal filter (*M*_r_ cutoff of 10,000; Millipore). Concentrations of the purified proteins were measured spectrophotometrically at 280 nm using the calculated extinction coefficients (calculated with http://web.expasy.org/protparam/). The purified proteins were stored in aliquots at −80°C.

### Purification of total protein from *Streptomyces* strains.

S. olindensis and S. lividans pUWL (apramycin) were incubated in 100 mL TS broth for 48 h at 30°C at 220 rpm. Five milliliters of the preculture was added to 100 mL of YEME (48) medium and incubated for a further 2 days at 30°C at 220 rpm. The cultures were stored on ice for 20 min and then centrifuged at 6,000 × *g* for 30 min at 4°C. The supernatant was discarded, and the pellet resuspended in lysis buffer (5 mL/g pellet) (50 mM Tris-HCl, pH 8, 500 mM NaCl, 10% glycerin [vol/vol], 1% Tween 20 [vol/vol], 20 mM imidazole, 10 mM β-mercaptoethanol, 0.5 mM PMSF, and 4 mg/mL lysozyme). The cells were ruptured with a French press under 10,000 lb/in^2^ pressure, and cell debris was removed by centrifugation at 4°C for 45 min at 15,000 rpm. The clear lysate was filtered (0.45 μm) for buffer exchange (25 mM KH_2_PO_4_, 100 mM NaCl, pH 8.3). The purified proteins were quantified and stored at the same concentration at −80°C.

### Peroxide and protein quantification.

The concentration of H_2_O_2_ stock solution (Sigma-Aldrich) was measured at 240 nm (ε240 nm = 43.6 M^−1^ cm^−1^) (52). Protein concentrations were determined spectrophotometrically at 280 nm; the molar absorption coefficients were calculated from the amino acid compositions (http://web.expasy.org/protparam/) (53). The calculated protein concentrations refer to those of monomers.

### FOX assay.

WT MPx and its mutant were reduced with 10× excess dithiothreitol (DTT) in buffer solution (250 mM Tris buffer, 500 mM NaCl, pH 8.0) and incubated for 30 min at room temperature. The ferrous oxidation of xylenol orange (FOX) assay (54) was used to determine the H_2_O_2_ consumption over time by the purified MPx or the C38S mutant and total proteins from S. olindensis and S. peucetius strains isolated during antibiotic production. All measurements were performed in three replicates for each treatment. The H_2_O_2_ concentration was calculated based on an H_2_O_2_ standard curve. In the case of purified MPx and mutant MPx, 100 μM was mixed with 100 μM H_2_O_2_ in 100 mM assay buffer, pH 8, at 30°C, and 10-μL amounts of the reaction mixture were taken after 15, 30, 45, 90, and 180 s and mixed with 490 μL of the FOX reaction mixture (100 μM xylenol orange, 250 μM ammonium ferrous sulfate, 100 mM sorbitol, and 25 mM H_2_SO_4_) and incubated for 30 min at room temperature in darkness. At the end of the reaction, the *A*_560_ was measured on a 96-well plate reader (FLUOstar Omega) as described elsewhere (54). To isolate total proteins from S. olindensis and S. lividans, cells were initially grown in TS broth at 30°C for 24 h. One percent preculture was used to inoculate the main R5M culture. This culture was grown at 30°C for protein extraction at 24, 48, and 72 h during the production of cosmomycin D for S. olindensis. Then, 100 mM H_2_O_2_ was added to equal concentrations of total proteins. Ten-microliter aliquots were taken after 100, 200, 300, 400, 500, and 600 s, as described elsewhere (54).

### Total RNA isolation and cDNA synthesis.

Total RNA of producer strain S. olindensis WT was extracted using RNAprotect bacterial reagent and the RNeasy minikit according to the manufacturer’s instructions (Qiagen). The frozen samples were ground in dry ice. Residual DNA was removed by treating with RNase-free DNase according to the manufacturer’s instructions (Qiagen). An aliquot of 1 μg of DNase-treated RNA was transcribed into cDNA using iScript reverse transcription supermix for reverse transcription (RT)-qPCR (Bio-Rad Laboratories, Inc.).

### qPCR analysis.

For qPCR, a Bio-Rad iQ5 thermocycler (Bio-Rad Laboratories, Inc.) was programmed for an initial denaturation of 5 min at 95°C, followed by 40 cycles of 5 s at 95°C and 15 s at 60°C. The specificity of the qPCR primers (Table S3) was evaluated by the melting curve, using a gradient of 55 to 95°C at 1°C each 30 s (Fig. S10). For each amplification reaction mixture volume, 2 μL of cDNA (50 ng), 10 μM each primer, and 10 μL of QuantiNova probe PCR kit (Qiagen) were used.

iQ5 Optical System software was used to determine the relative quantification of the target genes in comparison to the reference gene. The selection of an endogenous gene, to be used as a normalizer, was made by testing the cycle threshold (*C_T_*) values for the different described endogenous genes (Fig. S10). The *hdrB* housekeeping gene presented the lowest Δ*C_T_* and the highest amplification efficiency, resulting in its selection as the normalizer for gene expression analysis.

### Determination of MICs of anthracyclines.

Sterile 96-well plates were used to generate MIC values. Each well contained 100 μL of Bennett’s agar and doxorubicin or cosmomycin D with concentrations ranging from 0 to 512 μg/mL. Each well was inoculated with ~10^3^ Streptomyces spores. Plates were incubated overnight at 30°C. Growth was compared with that of the controls, S. peucetius, S. olindensis, and S. lividans TK24/pUWL (empty vector).

### Construction of SSNs and GNN.

Web tools of the Enzyme Function Initiative (EFI), available at https://efi.igb.illinois.edu/ (22), were used to generated sequence similarity networks (SSNs) from KDN80073.1 and the glutathione peroxidase Pfam family (PF00255). For the seed SSN obtained with the KDN80073.1 FASTA sequence, the option Maximum Number of Retrieve Sequences = 100 in the BLAST Retrieval Options tab was selected, and then the score threshold was adjusted to 65 using Percent Identity vs Alignment Score Box Plot in order to set up only the presence of nodes with identity equal to 70% or above.

For the initial submission of PF00255, all settings were kept at the default, and a first SSN was generated using a Score threshold of 50, which means each edge joins nodes with at least 55% similarity. The second SSN used a slightly higher score threshold of 60 (nodes are joined if they share about 61% or more identity). Using the EFI Genome Neighborhood Tool (EFI-GNT tab) of the same website, a genome neighborhood network (GNN) and genome neighborhood diagrams (GNDs) of the second SSN were obtained using as parameters Neighborhood Size = 20 and Cooccurrence Percentage Lower Limit = 20.
