# Supplementary material for: Mycothiol Peroxidase Activity as a Part of the Self-Resistance Mechanisms against the Antitumor Antibiotic Cosmomycin D
Source: Microbiol Spectr. 2022 May 5;10(3):e00493-22. doi: 10.1128/spectrum.00493-22 (PMC9241694; doi:10.1128/spectrum.00493-22)
Supplement: SUPPLEMENTAL FILE 1 — Supplemental material. Download spectrum.00493-22-s001.pdf, PDF file, 7.2 MB [file spectrum.00493-22-s001.pdf]

Supplementary Information for

Mycothiol peroxidase activity as a part of the self-resistance mechanisms against the antitumoral Cosmomycin D

Roger D. Castillo[1,2], Leandro M. Garrido[2], Brandán Pedre[3], Irina Helmle[1], Harald Gross[1,4], Bertolt Gust[1,4] and Gabriel Padilla[2]*

[1]Pharmaceutical Institute, Department of Pharmaceutical Biology, University of Tübingen, Auf der Morgenstelle 8, 72076 Tübingen, Germany

[2]Institute of Biomedical Sciences, University of São Paulo, São Paulo, CEP 005508-900, Brazil

[3]Division of Redox Regulation, DKFZ-ZMBH Alliance, German Cancer Research Center (DKFZ), 69120 Heidelberg, Germany

[4]German Center for Infection Research (DZIF), Partner Site Tübingen, 72076 Tübingen, Germany

This PDF file includes:

Supplementary Figures

Supplementary Tables

A

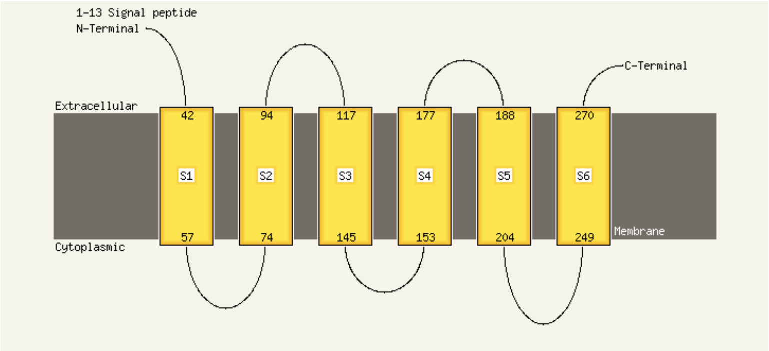

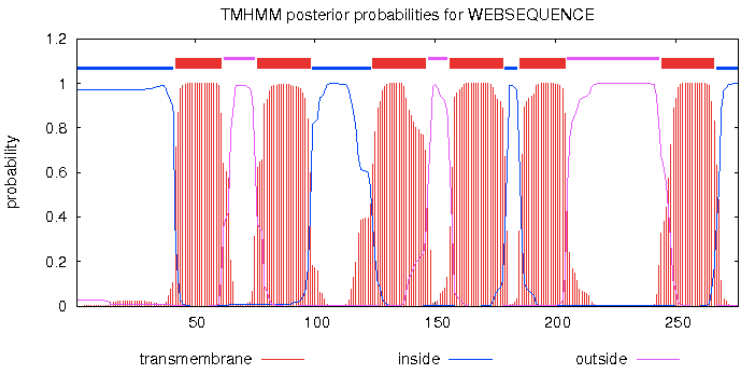

B

FIG S1 Distribution and probability of transmembrane helices encoded by *cos*J A) Prediction and topology of transmembrane helices using Phyre server v 2.0. B) Probability graph of transmembrane amino acids using TMHMM server v 2.0.

A.

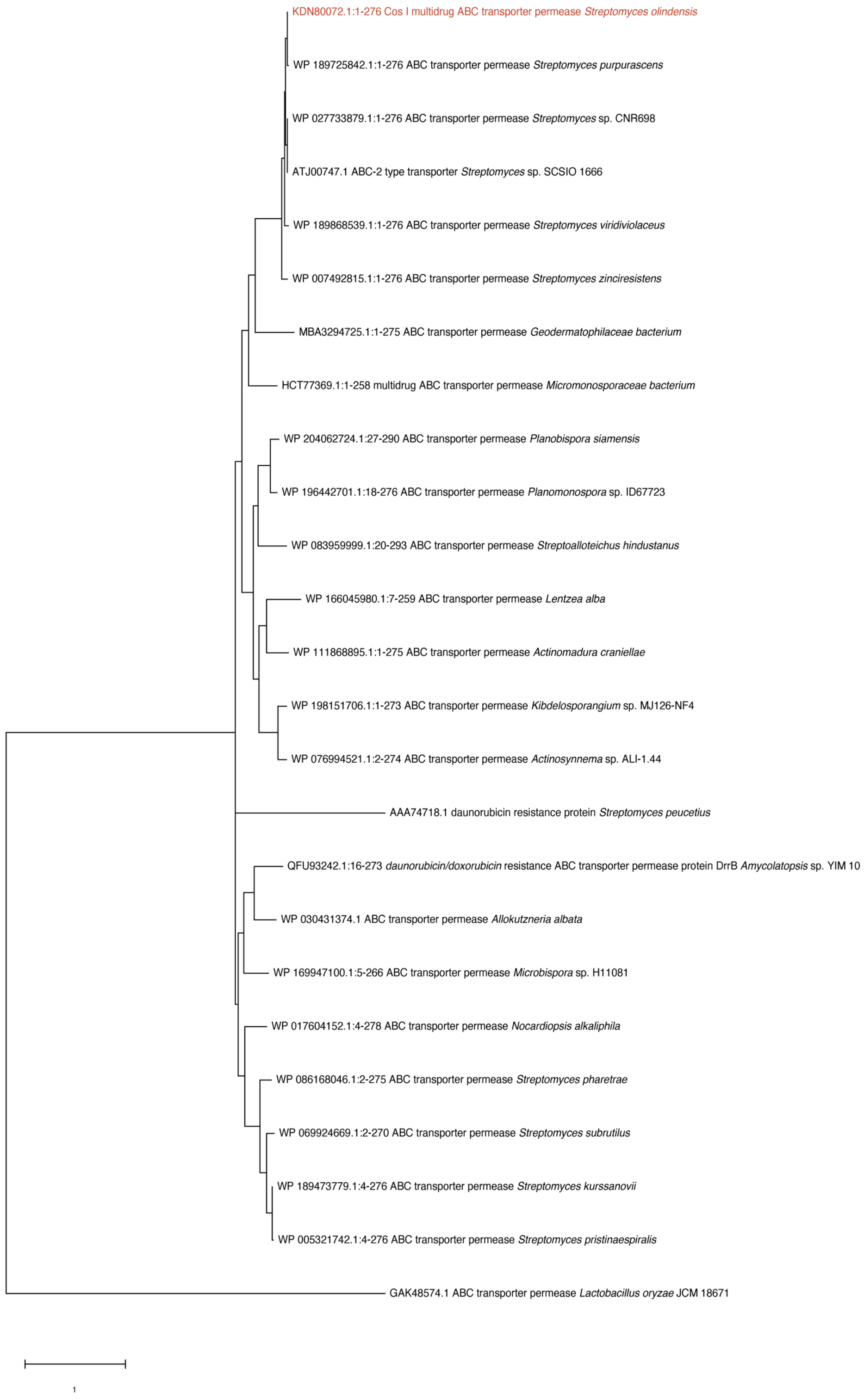

KDN80072.1:1-276 Cos I multidrug ABC transporter permease *Streptomyces olindensis*
WP 189725842.1:1-276 ABC transporter permease *Streptomyces purpurascens*
WP 027733879.1:1-276 ABC transporter permease *Streptomyces* sp. CNR698
ATJ00747.1 ABC-2 type transporter *Streptomyces* sp. SCSIO 1666
WP 189868539.1:1-276 ABC transporter permease *Streptomyces viridiviolaceus*
WP 007492815.1:1-276 ABC transporter permease *Streptomyces zinciresistens*
MBA3294725.1:1-275 ABC transporter permease *Geodermatophilaceae bacterium*
HCT77369.1:1-258 multidrug ABC transporter permease *Micromonosporaceae bacterium*
WP 204062724.1:27-290 ABC transporter permease *Planobispora siamensis*
WP 196442701.1:18-276 ABC transporter permease *Planomonospora* sp. ID67723
WP 083959999.1:20-293 ABC transporter permease *Streptoalloteichus hindustanus*
WP 166045980.1:7-259 ABC transporter permease *Lentzea alba*
WP 111868895.1:1-275 ABC transporter permease *Actinomadura craniellae*
WP 198151706.1:1-273 ABC transporter permease *Kibdelosporangium* sp. MJ126-NF4
WP 076994521.1:2-274 ABC transporter permease *Actinosynnema* sp. ALI-1.44
AAA74718.1 daunorubicin resistance protein *Streptomyces peucetius*
QFU93242.1:16-273 *daunorubicin/doxorubicin* resistance ABC transporter permease protein DrrB *Amycolatopsis* sp. YIM 10
WP 030431374.1 ABC transporter permease *Allokutzneria albata*
WP 169947100.1:5-266 ABC transporter permease *Microbispora* sp. H11081
WP 017604152.1:4-278 ABC transporter permease *Nocardiopsis alkaliphila*
WP 086168046.1:2-275 ABC transporter permease *Streptomyces pharetrae*
WP 069924669.1:2-270 ABC transporter permease *Streptomyces subrutilus*
WP 189473779.1:4-276 ABC transporter permease *Streptomyces kurssanovii*
WP 005321742.1:4-276 ABC transporter permease *Streptomyces pristinaespiralis*
GAK48574.1 ABC transporter permease *Lactobacillus oryzae* JCM 18671
1

B.

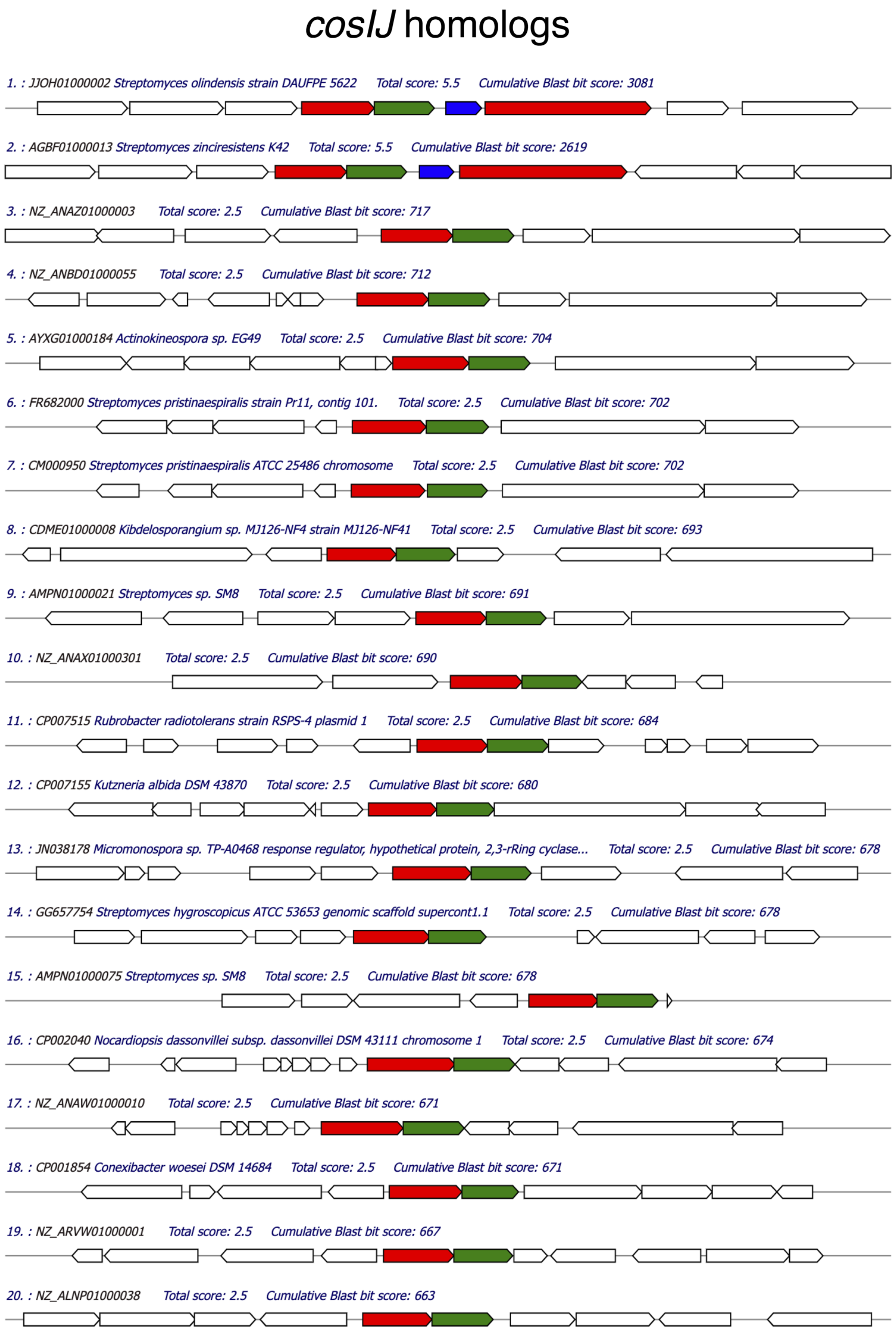
cosIJ homologs
1. : JJOH01000002 Streptomyces olindensis strain DAUFPE 5622 Total score: 5.5 Cumulative Blast bit score: 3081
2. : AGBF01000013 Streptomyces zincirisistens K42 Total score: 5.5 Cumulative Blast bit score: 2619
3. : NZ_ANAZ01000003 Total score: 2.5 Cumulative Blast bit score: 717
4. : NZ_ANBD01000055 Total score: 2.5 Cumulative Blast bit score: 712
5. : AYXG01000184 Actinokineospora sp. EG49 Total score: 2.5 Cumulative Blast bit score: 704
6. : FR682000 Streptomyces pristinaespiralis strain Pr11, contig 101. Total score: 2.5 Cumulative Blast bit score: 702
7. : CM000950 Streptomyces pristinaespiralis ATCC 25486 chromosome Total score: 2.5 Cumulative Blast bit score: 702
8. : CDME01000008 Kibdelosporangium sp. MJ126-NF4 strain MJ126-NF41 Total score: 2.5 Cumulative Blast bit score: 693
9. : AMPN01000021 Streptomyces sp. SM8 Total score: 2.5 Cumulative Blast bit score: 691
10. : NZ_ANAX01000301 Total score: 2.5 Cumulative Blast bit score: 690
11. : CP007515 Rubrobacter radiotolerans strain RSPS-4 plasmid 1 Total score: 2.5 Cumulative Blast bit score: 684
12. : CP007155 Kutzneria albida DSM 43870 Total score: 2.5 Cumulative Blast bit score: 680
13. : JN038178 Micromonospora sp. TP-A0468 response regulator, hypothetical protein, 2,3-rRing cyclase... Total score: 2.5 Cumulative Blast bit score: 678
14. : GG657754 Streptomyces hygroscopicus ATCC 53653 genomic scaffold supercont1.1 Total score: 2.5 Cumulative Blast bit score: 678
15. : AMPN01000075 Streptomyces sp. SM8 Total score: 2.5 Cumulative Blast bit score: 678
16. : CP002040 Nocardiopsis dassonvillei subsp. dassonvillei DSM 43111 chromosome 1 Total score: 2.5 Cumulative Blast bit score: 674
17. : NZ_ANAW01000010 Total score: 2.5 Cumulative Blast bit score: 671
18. : CP001854 Conexibacter woesei DSM 14684 Total score: 2.5 Cumulative Blast bit score: 671
19. : NZ_ARVW01000001 Total score: 2.5 Cumulative Blast bit score: 667
20. : NZ_ALNP01000038 Total score: 2.5 Cumulative Blast bit score: 663

FIG 2 A. Phylogenetic analysis of the *cosI* gene. The maximum likelihood tree is based on NCBI database, MultiGeneBlast and performed with MEGA X. *Lactobacillus oryzae JCM 18671* (GAK48574.1) was used as the outgroup. Bootstrap analysis (performed 1,000 times). Scale bar represents 1 amino acid substitutions per site. **B.** MultiGeneBlast results using *cosI, cosJ, cosP,* and *cosU* as a template. *cosI, cosJ* (ABC transporter) are shown in red and green

A.

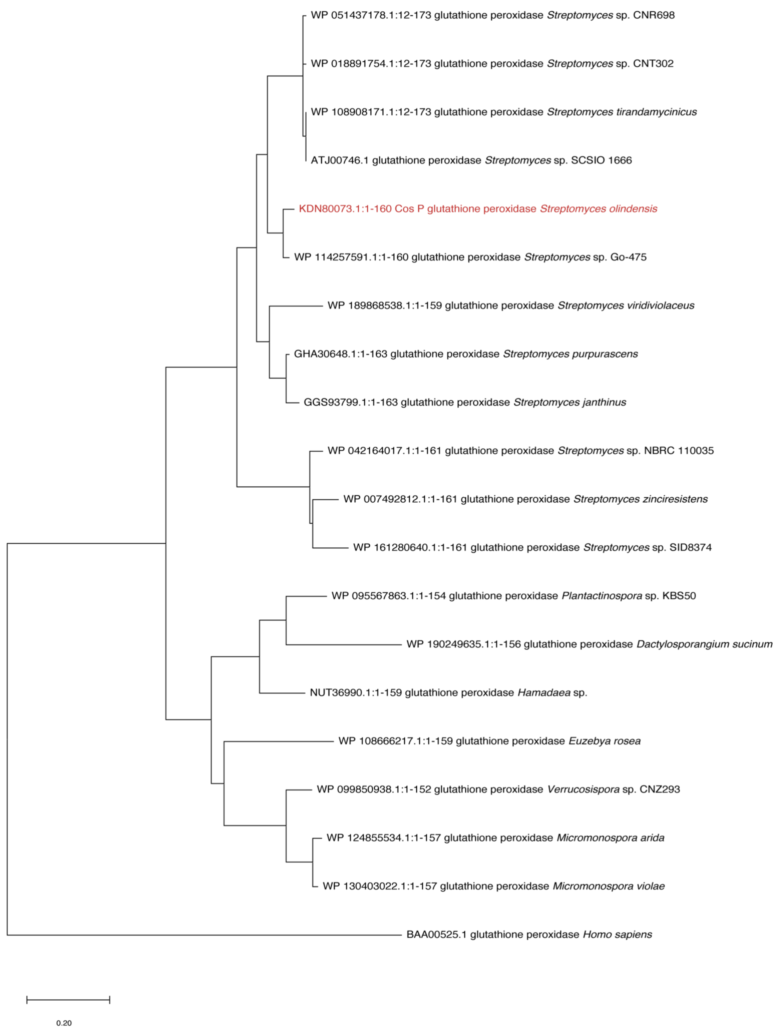

B.

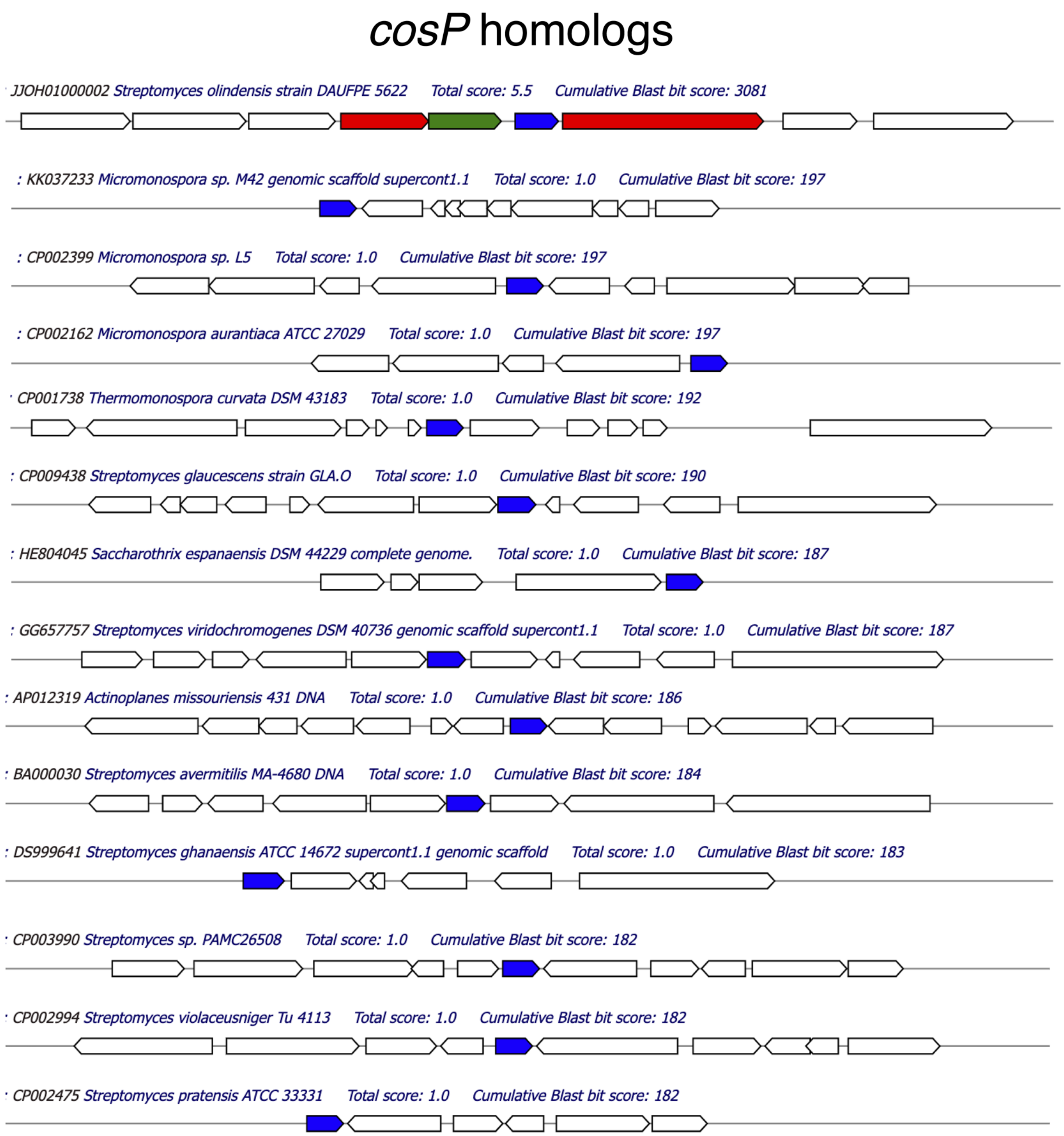
cosP homologs
JJOH01000002 Streptomyces olindensis strain DAUFPE 5622 Total score: 5.5 Cumulative Blast bit score: 3081
: KK037233 Micromonospora sp. M42 genomic scaffold supercont1.1 Total score: 1.0 Cumulative Blast bit score: 197
: CP002399 Micromonospora sp. L5 Total score: 1.0 Cumulative Blast bit score: 197
: CP002162 Micromonospora aurantiaca ATCC 27029 Total score: 1.0 Cumulative Blast bit score: 197
CP001738 Thermomonospora curvata DSM 43183 Total score: 1.0 Cumulative Blast bit score: 192
: CP009438 Streptomyces glaucescens strain GLA.O Total score: 1.0 Cumulative Blast bit score: 190
: HE804045 Saccharothrix espanaensis DSM 44229 complete genome. Total score: 1.0 Cumulative Blast bit score: 187
: GG657757 Streptomyces viridochromogenes DSM 40736 genomic scaffold supercont1.1 Total score: 1.0 Cumulative Blast bit score: 187
: AP012319 Actinoplanes missouriensis 431 DNA Total score: 1.0 Cumulative Blast bit score: 186
: BA000030 Streptomyces avermitilis MA-4680 DNA Total score: 1.0 Cumulative Blast bit score: 184
: DS999641 Streptomyces ghanaensis ATCC 14672 supercont1.1 genomic scaffold Total score: 1.0 Cumulative Blast bit score: 183
CP003990 Streptomyces sp. PAMC26508 Total score: 1.0 Cumulative Blast bit score: 182
CP002994 Streptomyces violaceusniger Tu 4113 Total score: 1.0 Cumulative Blast bit score: 182
CP002475 Streptomyces pratensis ATCC 33331 Total score: 1.0 Cumulative Blast bit score: 182

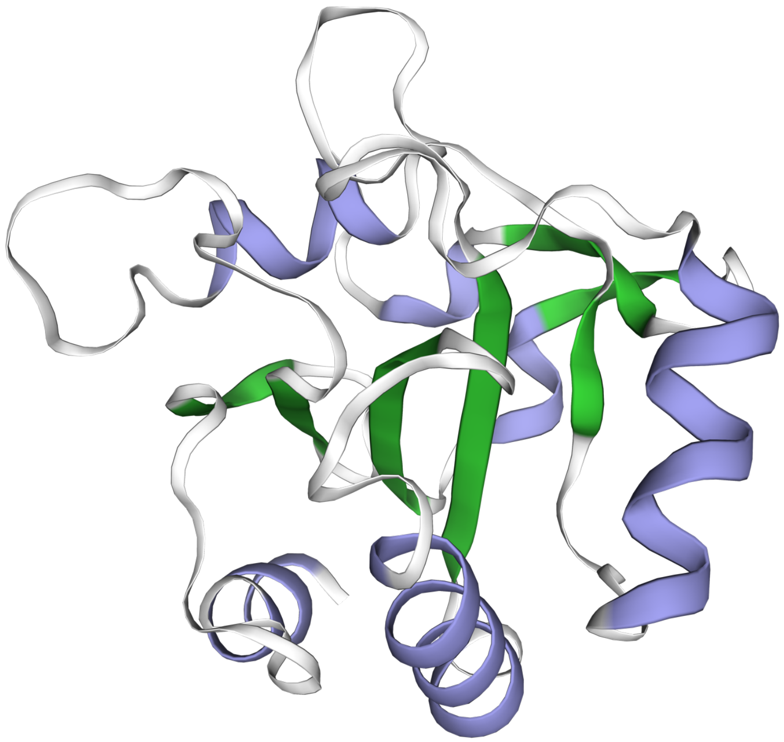

FIG 3 A Phylogenetic analysis of the *cosP* gene. The maximum likelihood tree is based on NCBI database, MultiGeneBlast and performed with MEGA X. *Homo sapiens* (BAA00525.1) was used as the outgroup. Bootstrap analysis (performed 1,000 times). Scale bar represents 0.20 amino acid substitutions per site. **B.** MultiGeneBlast results using *cosI, cosJ, cosP,* and *cosU* as a template. *cosP* (Mpx) homologs are shown in blue **C.** Structure protein model of the Cos P. Model based on crystal structure of *Schistosoma mansoni* glutathione peroxidase from SWISS- MODEL database tool (https://swissmodel.expasy.org).

A.

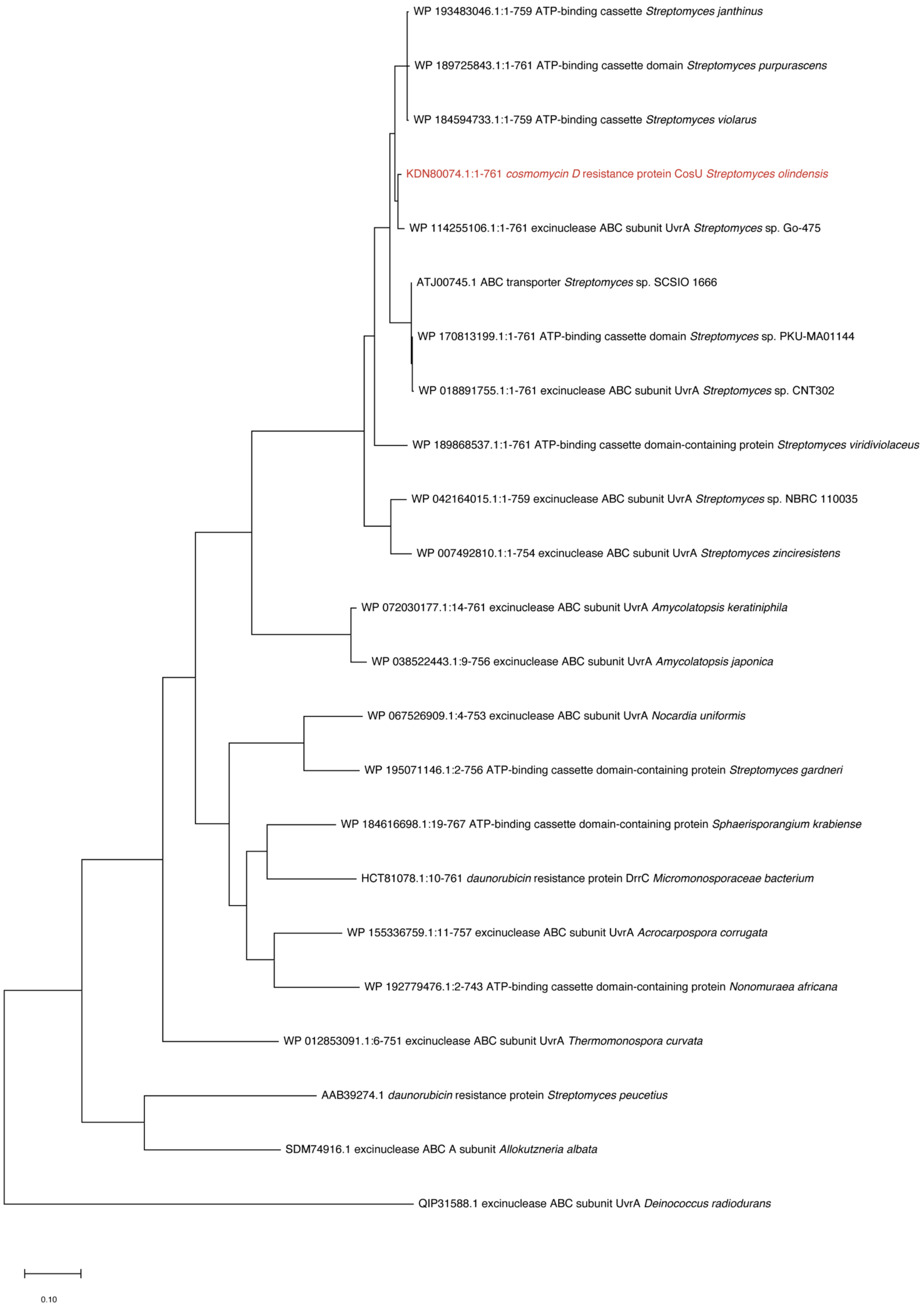
WP 193483046.1:1-759 ATP-binding cassette Streptomyces janthinus
WP 189725843.1:1-761 ATP-binding cassette domain Streptomyces purpurascens
WP 184594733.1:1-759 ATP-binding cassette Streptomyces violarus
KDN80074.1:1-761 cosmomycin D resistance protein CosU Streptomyces olindensis
WP 114255106.1:1-761 excinuclease ABC subunit UvrA Streptomyces sp. Go-475
ATJ00745.1 ABC transporter Streptomyces sp. SCSIO 1666
WP 170813199.1:1-761 ATP-binding cassette domain Streptomyces sp. PKU-MA01144
WP 018891755.1:1-761 excinuclease ABC subunit UvrA Streptomyces sp. CNT302
WP 189868537.1:1-761 ATP-binding cassette domain-containing protein Streptomyces viridiviolaceus
WP 042164015.1:1-759 excinuclease ABC subunit UvrA Streptomyces sp. NBRC 110035
WP 007492810.1:1-754 excinuclease ABC subunit UvrA Streptomyces zinciresistens
WP 072030177.1:14-761 excinuclease ABC subunit UvrA Amycolatopsis keratiniphila
WP 038522443.1:9-756 excinuclease ABC subunit UvrA Amycolatopsis japonica
WP 067526909.1:4-753 excinuclease ABC subunit UvrA Nocardia uniformis
WP 195071146.1:2-756 ATP-binding cassette domain-containing protein Streptomyces gardneri
WP 184616698.1:19-767 ATP-binding cassette domain-containing protein Sphaerisporangium krabiense
HCT81078.1:10-761 daunorubicin resistance protein DrrC Micromonosporaceae bacterium
WP 155336759.1:11-757 excinuclease ABC subunit UvrA Acrocarpospora corrugata
WP 192779476.1:2-743 ATP-binding cassette domain-containing protein Nonomuraea africana
WP 012853091.1:6-751 excinuclease ABC subunit UvrA Thermomonospora curvata
AAB39274.1 daunorubicin resistance protein Streptomyces peucetius
SDM74916.1 excinuclease ABC A subunit Allokutzneria albata
QIP31588.1 excinuclease ABC subunit UvrA Deinococcus radiodurans
0.10

B.

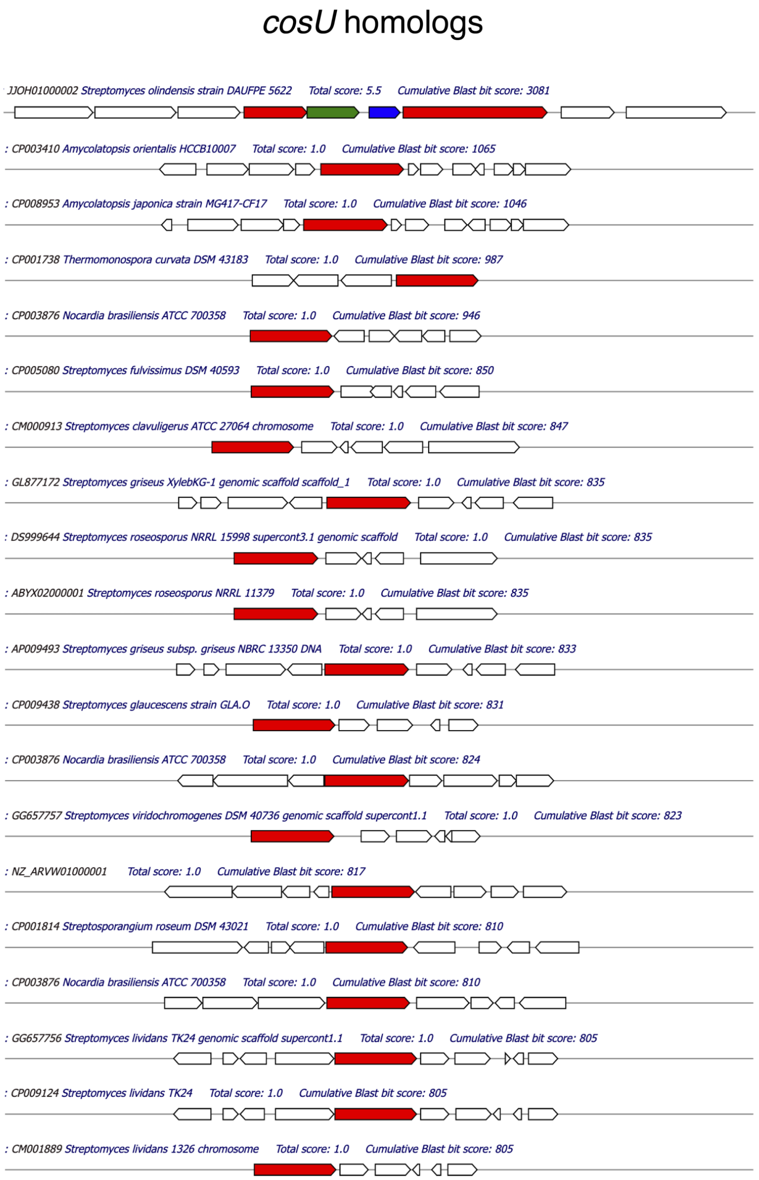

FIG S4 Phylogenetic analysis of the *cosU* gene. The maximum likelihood tree is based on NCBI database, MultiGeneBlast and performed with MEGA X. *Deniococcus radiodurans* (QIP31588.1) was used as the outgroup. Bootstrap analysis (performed 1,000 times). Scale bar represents 0.10 amino acid substitutions per site. **B.** MultiGeneBlast results using *cosI, cosJ, cosP,* and *cosU* as a template. *cosU* (UvrA like protein) homologs are shown in red

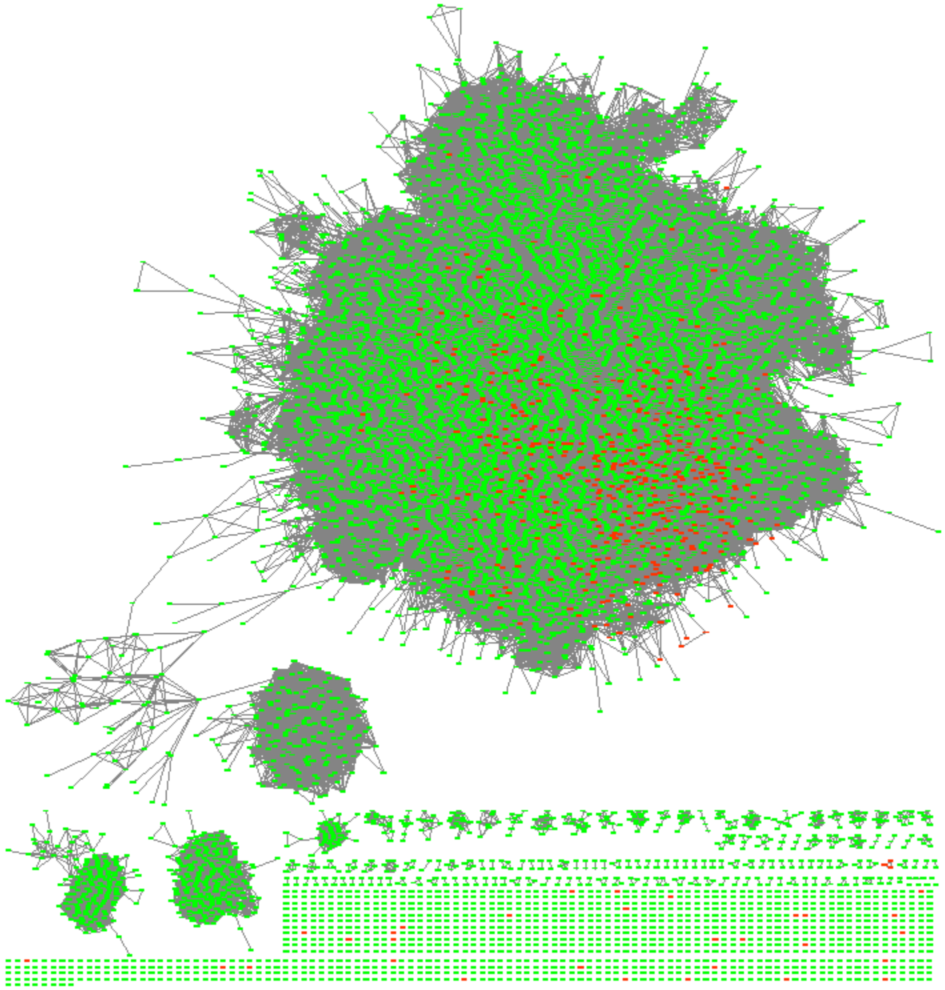

FIG S5 First Gpx PFAM Family PF00255 SSN. Identity threshold around 55%. Red nodes denote actinobacteria peroxidases, green nodes represent all the other prokaryotic and eukaryotic members of the family. The biggest cluster (cluster 1) comprises 39799 proteins including prokaryotic and eukaryotic known Gpx like *E. coli* BtuE (P06610) and *S. cerevisae* Gpx1 (P36014), as well as most actinobacteria proteins.

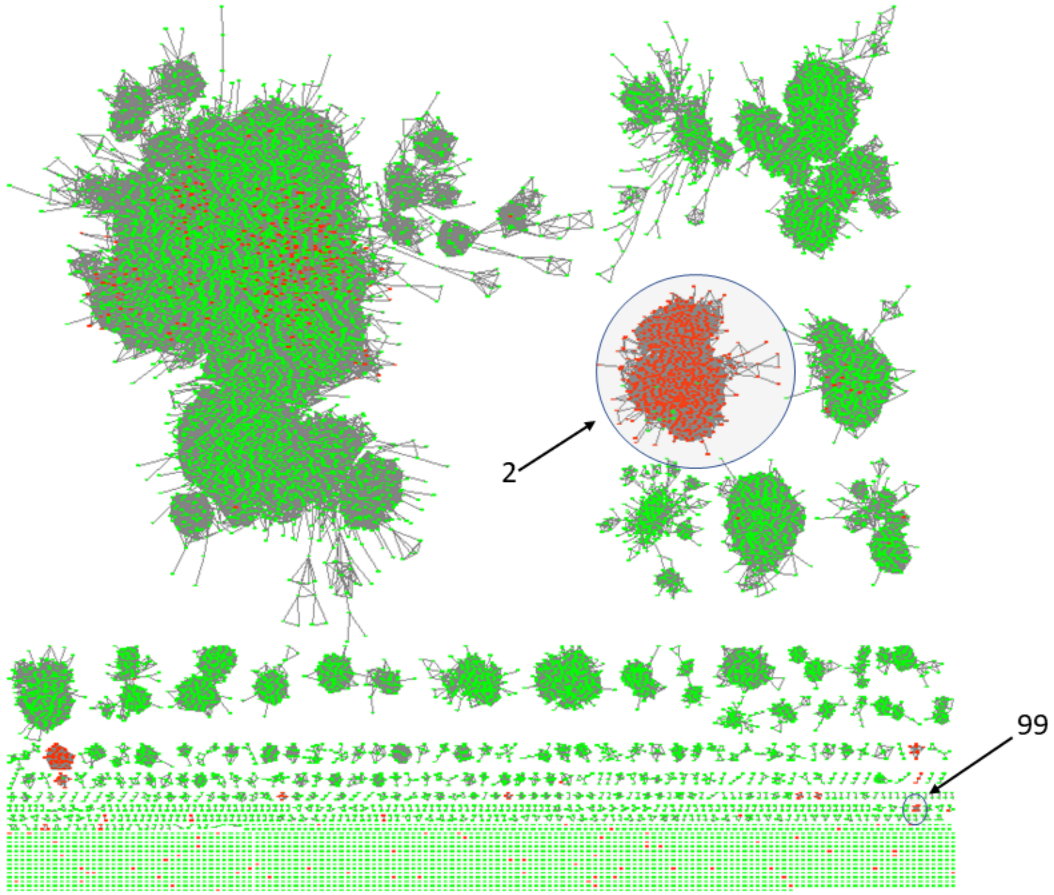

FIG S6 Second Gpx PFAM Family PF00255 SSN. Identity threshold around 61%. Red nodes denote actinobacteria members of family, green nodes represents all the other prokaryotic and eukaryotic members of the family. Cluster 2 (where CAB88451.1, WP_010985209.1, WP_100106014.1 and EFL32854.1 group with most actinobacteria peroxidases) and 99 (where lies relatives of KDN800793.1 are highlighted.

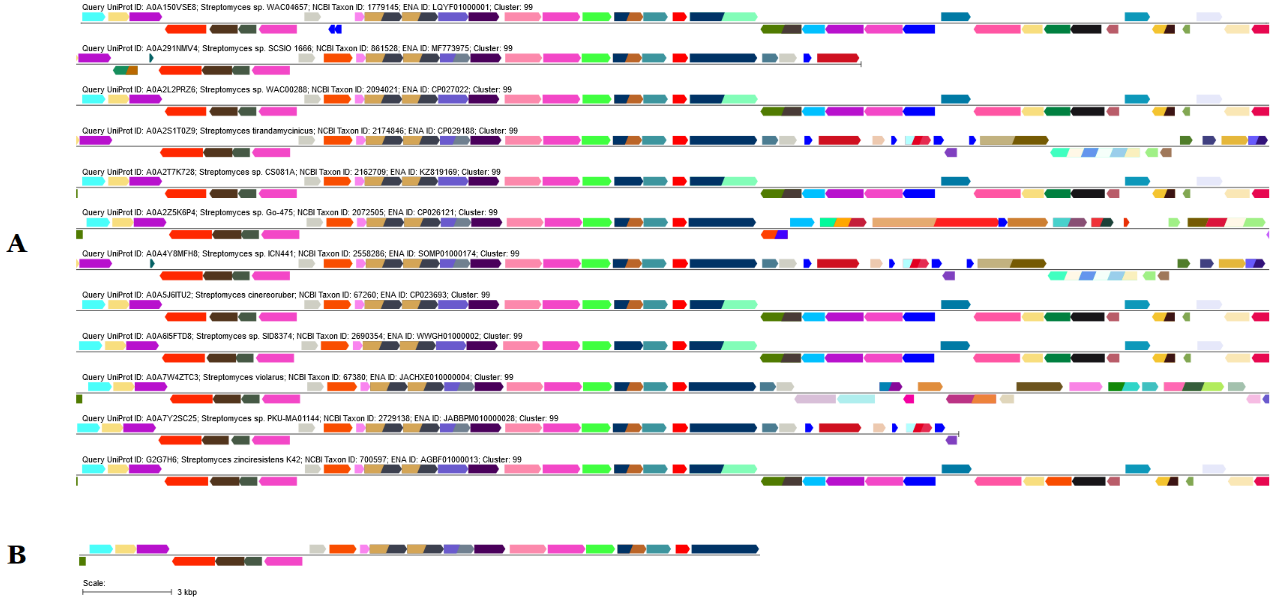

FIG S7 Genome Neighborhood Diagram of cluster 99 (2nd PF00255 SSN). A-Alignment of genome context alongside of *cosP* orthologs of all cluster 99 members; B: Cosmomycin BGC cluster region of *S. olindensis* corresponding to GND of part A. Orthologs of *cosP* orthologs (painted in red) are at the center of the GND.

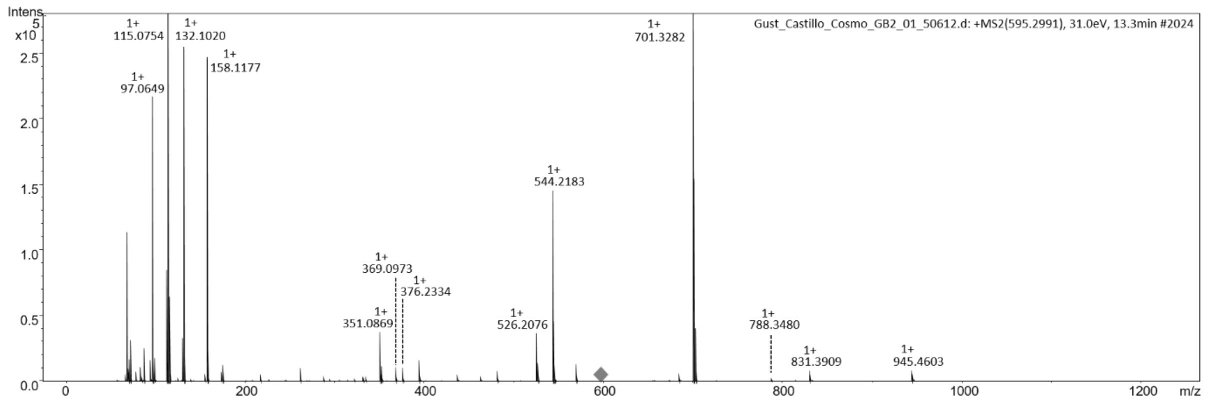

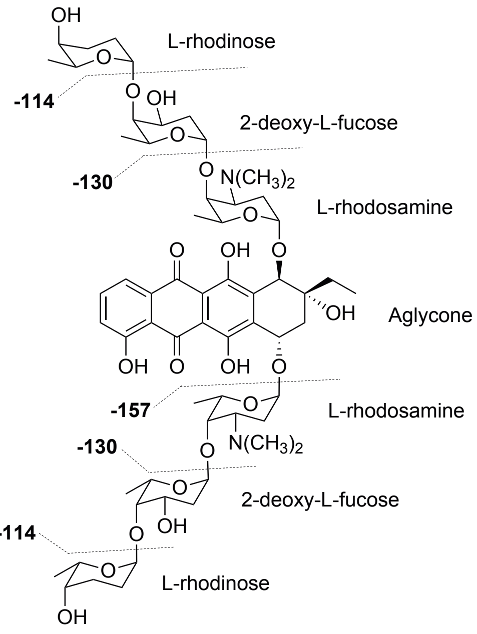

cosmomycin D

FIG. S8: ESI-MS/MS (MS^2) of cosmomycin D (top). The dotted lines were drawn to extend the intensities of the observed peaks. Bottom scheme shows the key fragments corresponding to the structure of CosD and were assigned on the structure.

TABLE S1: With tandem MS/MS, pure cosmomycin D was fragmented yielding several daughter ions in the mass spectrum. Theoretical and observed mass differences between these assignments were negligible.

Fragments	Elemental composition	Relative Double Bond (Rdb)	Theoretical Mass	Experimental Mass *(m/z)*	Error [ppm]
$[M+H]^{2+}$	$C_{60}H_{90}N_2O_{22}$	18	595.2987	595.2991	0.67
$[M-114-130+H]^+$	$C_{48}H_{69}N_2O_{17}$	16	945.4596	945.4588	-0.85
$[M-2x114-130+H]^+$	$C_{42}H_{59}N_2O_{15}$	15	831.3915	831.3909	-0.72
$[M-114-130-157+H]^+$	$C_{40}H_{54}NO_{15}$	15	788.3493	788.3480	-1.65
$[M-2x114-2x130+H]^+$	$C_{36}H_{49}N_2O_{12}$	14	701.3286	701.3282	-0.57
$[M-2x114-2x130-157+H]^+$	$C_{28}H_{34}NO_{10}$	13	544.2183	544.2183	0.00
$[M-2x114-2x130-157-H_2O+H]^+$	$C_{28}H_{32}NO_9$	14	526.2077	526.2076	-0.19
Aglycone – H_2O	$C_{20}H_{17}O_7$	13	369.0974	369.0973	-0.27
Agylcone – $2xH_2O$	$C_{20}H_{15}O_6$	14	351.0869	351.0869	0.00
Sugar 1, 2 and 3 – $2xCH_3$	$C_{18}H_{34}NO_7$	3	376.2335	376.2334	-0.26
Sugar 3/3‘	$C_8H_{15}NO_2$	2	158.1181	158.1177	-2-53
Sugar 3/3‘ – $2xCH_3$	$C_6H_{14}NO_2$	1	132.1025	132.1020	-3.78
Sugar 2/2‘	$C_6H_{11}O_3$	2	131.0708	131.0704	-3.05
Sugar 1/1‘	$C_6H_{11}O_2$	2	115.0759	115.0754	-4.34
Sugar 1/1‘ – H_2O	C_6H_9O	3	97.0653	97.0649	-4.12

Melt Curve Peak Chart (-dF/dt vs T) **PCR Amp/Cycle Chart**

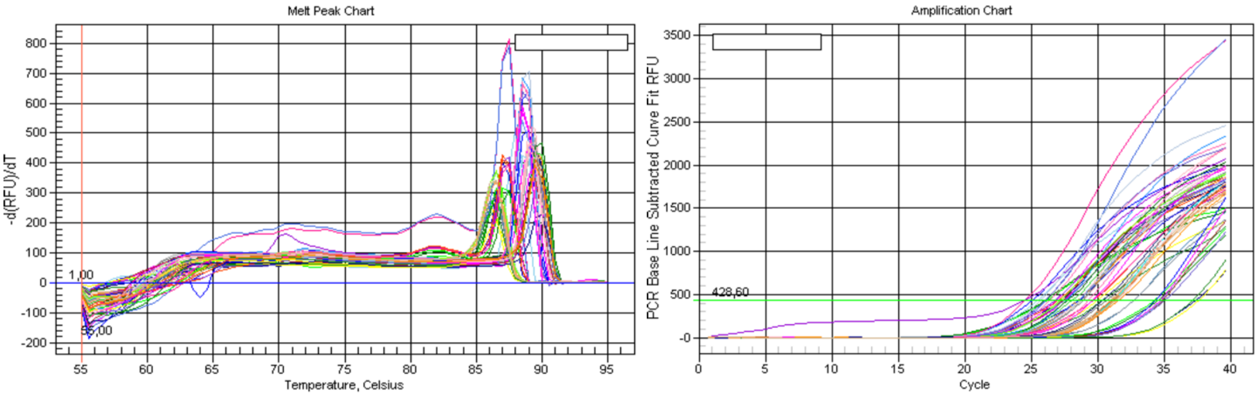

FIG S9 RT- qPCR (Melt Curve and PCR Amp/Cycle) of *cos*I, *cos*J, *cos*P, *cos*U and *Hrdb* during the production and non-production of COSD in *S. olindensis* strain. The analysis includes 3 biological replicates and 2 technical replicates for each treatment. In the same way, positive and negative controls were carried out to evaluate gDNA contamination and primer dimers with the melting curve.

A

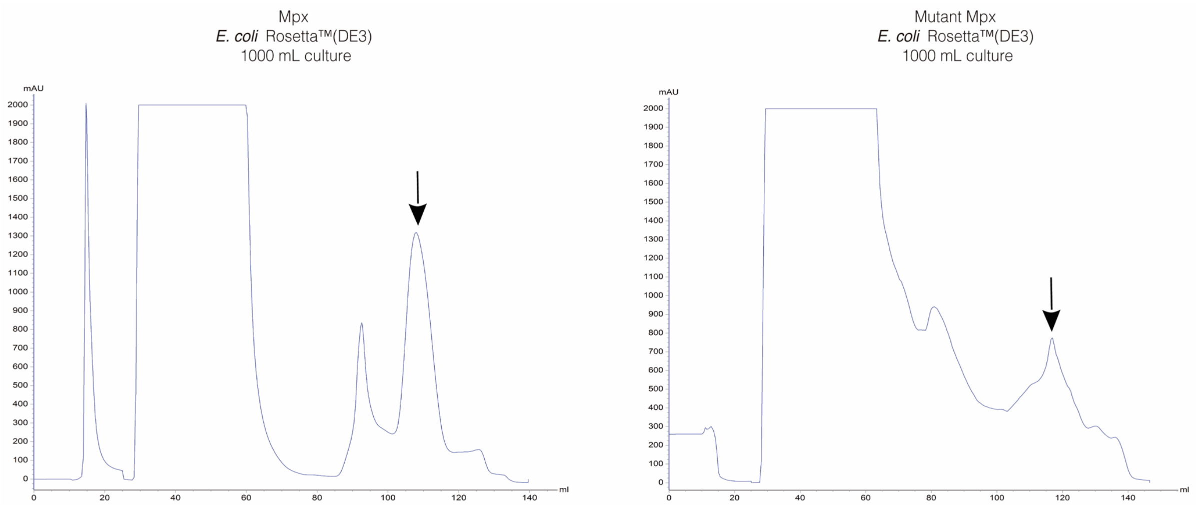

B

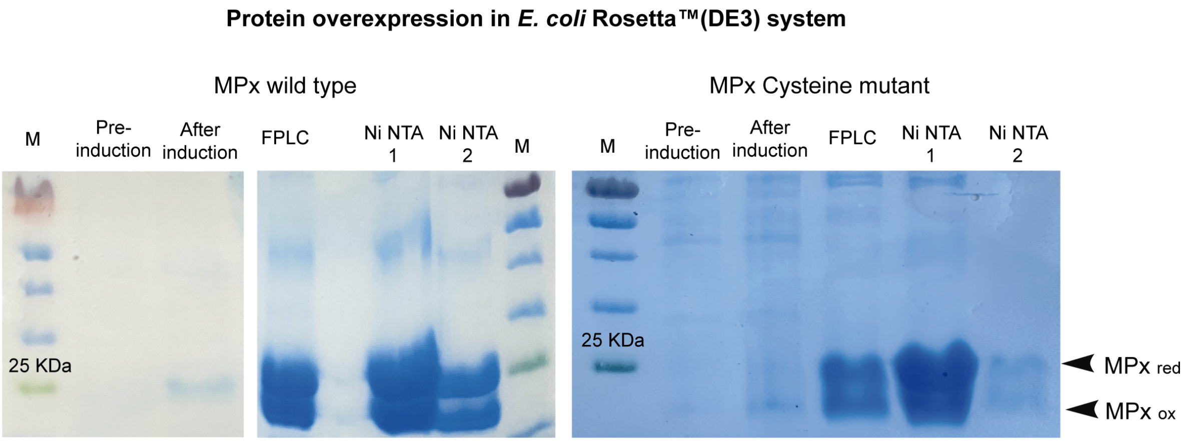

FIG S10 A. FPLC Chromatogram of the Mpx and its mutant Mpx C38S in *E. coli* Rossetta (DE3) as a host B.Fig X. SDS-PAGE of purified Mpx and MPx Cys28Ser mutant obtained by FPLC and Ni-NTA.

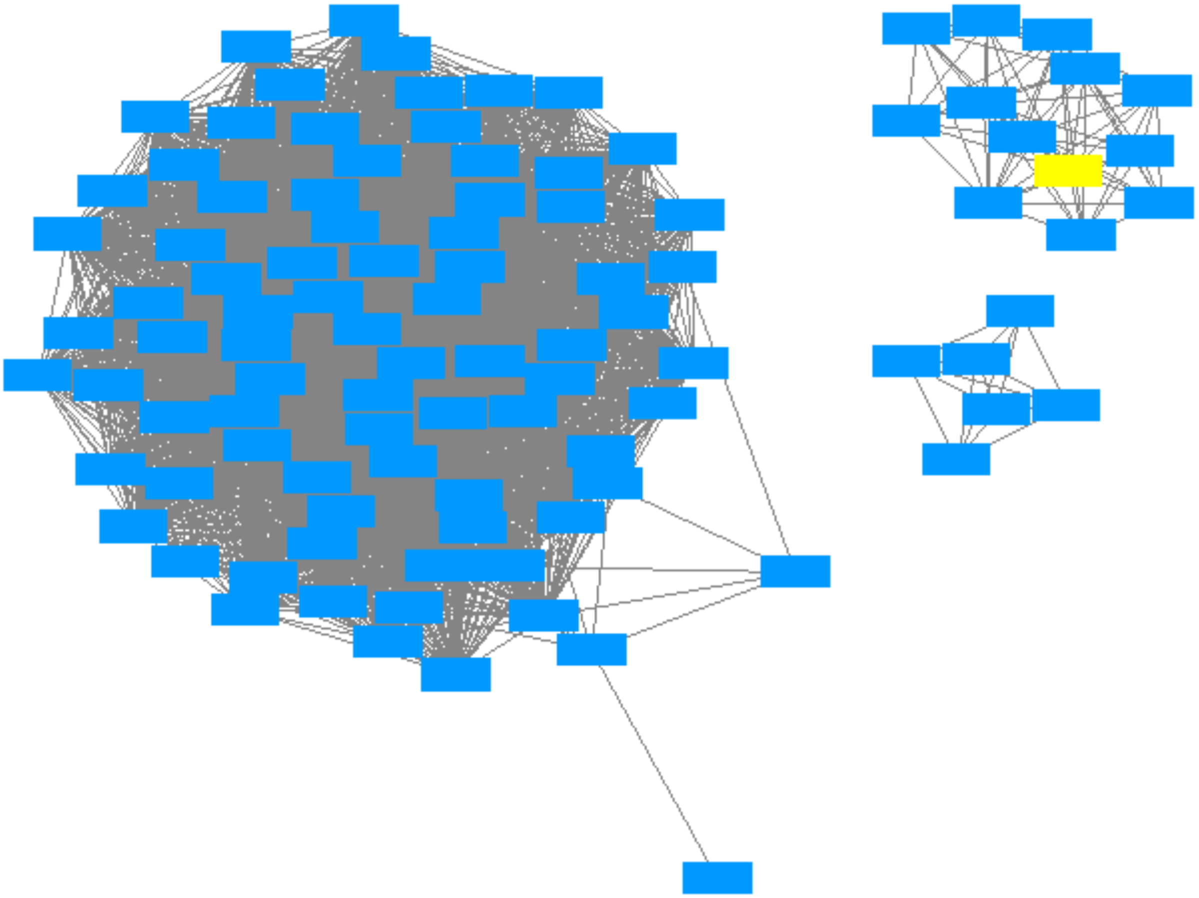

Fig S11: Seed SSN of KDN80073.3. SSN created to found close relatives of CosP (Highlighted in Yellow), to further compare the distribution of them in SSNs of PF00255.

TABLE S2 Cluster 2 of KDN80073,1 seed SSN

**Uniprot Acess number**	**NCBI protein ID**	**genome locus_tag**	**Description**	**Species**
**N/A**	**KDN80073.1**	**DF19_23570**	**Glutathione peroxidase (inputed sequence)**	***Streptomyces olindensis***

**A0A150VSE8**	**KYG53407.1**	**AWI43_02085**	**Glutathione peroxidase**	***Streptomyces sp.* WAC04657**
**A0A291NMV4**	**ATJ00746**	**MF773975.1**	**Glutathione peroxidase**	***Streptomyces sp.* SCSIO 1666**
**A0A2L2PRZ6**	**AVH94681.1**	**C5L38_06145**	**Glutathione peroxidase**	***Streptomyces sp.* WAC00288**
**A0A2S1T0Z9**	**AWI32167.1**	**DDW44_27735**	**Glutathione peroxidase**	***Streptomyces tirandamycinicus***
**A0A2T7K728**	**PVC77314.1**	**DBP18_02655**	**Glutathione peroxidase**	***Streptomyces sp.* CS081A**
**A0A2Z5K6P4**	**AXE88381.1**	**C1703_25560**	**Glutathione peroxidase**	***Streptomyces sp*. Go-475**
**A0A4Y8MFH8**	**TFE36212.1**	**E3E14_30925**	**Glutathione peroxidase**	***Streptomyces sp.* ICN441**
**A0A5J6ITU2**	**QEV35549.1**	**CP977_28010**	**Glutathione peroxidase**	***Streptomyces cinereoruber***
**A0A6I5FTD8**	**WP_161280640.1**	**GTY67_27700**	**Glutathione peroxidase**	**Streptomyces sp. SID8374**
**A0A7W4ZTC3**	**MBB3078325 ***	**FHS41_004832**	**Glutathione peroxidase**	***Streptomyces violarus***
**A0A7Y2SC25**	**NNJ05130.1**	**HHX38_13425**	**Glutathione peroxidase**	***Streptomyces sp.* PKU-MA01144**

**G2G7H6**	**EGX60546.1**	**SZN_07193**	**Glutathione peroxidase**	***Streptomyces zinciresistens* K42**

* Identical to MBB3078325 from another strain of *S. violarus*

TABLE S3 Primers used for plasmid construction (F: forward, R: reverse) confirmation (Test) and quantitative PCR (q)

**Product**	**Primer**
pUWL *cos*IJ F	AAAAA AAGCTTATGGGCGGCGCCGACCTGG
pUWL *cos*IJ R	AAAAA GAATTCTTACCTGCTCAGGGCCCGGT
pUWL *cos*P F	AAAAA AAGCTTATGACCGTCTTCGACATCGC
pUWL *cos*P R	AAAAA GAATTCTCACTGCACCTGCGCGC
pUWL *cos*U F	AAAAAAAGCTTGTGAACCAGACGCCGGTCAC
pUWL *cos*U R	AAAAA GAATTCGCCTCTACCTGGCCCAGTAG
pHIS8 *cos*P F	AAAAAGAATTCATGACCGTCTTCGACATCGC
pHIS8 *cos*P R	AAAAAAAGCTTTCACTGCACCTGCGCGC
Site mutagenesis cosP F	AACGTCGCCTCCCGCTCCGCCCTGG
Site mutagenesis cosP R	CTGGCCGGCCAGGGCGGAGC
Test pUWL test F	ACGCCTGGTCGATGTCGGAC
Test pUWL test R	GCTCTTCCGCTTCCTCGCTC
Test pHIS8 T7 test F	GGTTATGCTAGTTATTGCTCAGC
Test pHIS8 T7 test R	ATCTCGATCCCGCGAAATTAATAC
q *cosI* F	GATCCAGACCGAAGCTCTGA

q *cosI* R	TTCCCTCGTCGTACTTCAGC
q *cosJ* F	ATCCTGATGGTGCTGCTGTT
q *cosJ* R	AGCATGTTGACGATGCTCTG
q *cosP* F	CCTGCTCATCGTCAACGTC
q *cosP* R	GTGAGGGGGAACGTGATCT
q *cosU* F	CCAGACCTGGCTCTACTCCTT
q *cosU R*	TAGTCCGCGAGCTTCTTCTC
q HrdB F	TGCACATGGTCGAGGTCATC
q HrdB R	TCATGTCGAGTTCCTTGGCC

TABLE S4 Plasmids used in this study

pHis8	Expression vector; pET-28a (+) (Merck) derivative, N-terminal His8-tag and C-terminal His6-tag, T7 promotor, Kan^R	(Jez, Bowman, and Noel 2001)
pUWL-apra-oriT	*E. coli* - *Streptomyces* shuttle vector, pUWL201 derivative, *ermE** promotor, colE1, rep; Amp^R, $Apra^R$	Andreas Günther, Universität Frankfurt
pRCWL04	PCR product from gDNA of *Streptomyces olindensis* comprising *cos*IJ flanked by *Hind*III/*EcoRI* restriction sites in pUWL-apra-oriT, $Apra^R$	This study
pRCWL05	PCR product from gDNA of *Streptomyces olindensis* comprising *cos*P flanked by *Hind*III/*EcoRI* restriction	This study

	sites in pUWL-apra-oriT, Apra^R	
pRCWL06	PCR product from gDNA of *Streptomyces olindensis* comprising *cos*U flanked by *Hind*III/*EcoRI* restriction sites in pUWL-apra-oriT, Apra^R	This study
pRCIS04	PCR product from gDNA of *Streptomyces olindensis* comprising *cosP* flanked by *EcoR*I / *Hind*III restriction sites in pHis8, Kan^R	This study
pRCIS04M	Site mutagenesis from pRCIS04 comprising *cosP* C38S flanked by *EcoR*I / *Hind*III restriction sites in pHis8, Kan^R	This study
